# How to support the application of multiple criteria decision analysis? Let us start with a comprehensive taxonomy☆

**DOI:** 10.1016/j.omega.2020.102261

**Published:** 2020

**Authors:** Marco Cinelli, Miłosz Kadziński, Michael Gonzalez, Roman Słowiński

**Affiliations:** a Institute of Computing Science, Poznań University of Technology, Piotrowo 2, 60-965 Poznań, Poland; b Environmental Decision Analytics Branch, Land Remediation and Technology Division, Center for Environmental Solutions and Emergency Response, Office of Research and Development, U.S. Environmental Protection Agency, 26 West Martin Luther King Dr., Cincinnati, 45268, OH, United States; c Systems Research Institute, Polish Academy of Sciences, Newelska 6, 01-447 Warsaw, Poland

**Keywords:** Decision making, Multiple criteria, Taxonomy, Decision support system, MCDA, Method recommendation

## Abstract

Decision making is a complex task that involves a multitude of perspectives, constraints, and variables. Multiple Criteria Decision Analysis (MCDA) is a process that has been used for several decades to support decision making. It includes a series of steps that systematically help Decision Maker(s) (DM(s)) and stakeholders in structuring a decision making problem, identifying their preferences, and building a decision recommendation consistent with those preferences. Over the last decades, many studies have demonstrated the conduct of the MCDA process and how to select an MCDA method. Until now, there has not been a review of these studies, nor a proposal of a unified and comprehensive high-level representation of the MCDA process characteristics (i.e., features), which is the goal of this paper. We introduce a review of the research that defines how to conduct the MCDA process, compares MCDA methods, and presents Decision Support Systems (DSSs) to recommend a relevant MCDA method or a subset of methods. We then synthesize this research into a taxonomy of characteristics of the MCDA process, grouped into three main phases, (i) problem formulation, (ii) construction of the decision recommendation, and (iii) qualitative features and technical support. Each of these phases includes a subset of the 10 characteristics that helps the analyst implementing the MCDA process, while also being aware of the implication of these choices at each step. By showing how decision making can be split into manageable and justifiable steps, we reduce the risk of overwhelming the analyst, as well as the DMs/stakeholders during the MCDA process. A questioning strategy is also proposed to demonstrate how to apply the taxonomy to map MCDA methods and select the most relevant one(s) using real case studies. Additionally, we show how the DSSs for MCDA method recommendation can be grouped into three main clusters. This proposal can enhance a traceable and categorizable development of such systems.

## 1. Introduction

This article reviews the features that have been considered when leading multiple criteria-based decision aiding. It also lays a path for improving the structured and justifiable application of the Multiple Criteria Decision Analysis/Aiding (MCDA) process, as well as the selection of relevant MCDA method(s) for assessing a set of alternatives. Alternatives are at the basis of our daily decision making, spanning our personal (e.g., shirt to wear, meal to choose) and professional life. They include, e.g., different policy options, technologies for energy production or land remediation, new chemicals and products, sustainable development plans, and energy scenarios. The list of alternatives we deal with every day is endless because most of the decision making processes involve comparisons to select one or a subset from a larger pool. To reach the decision, the alternatives must be evaluated with respect to one or multiple evaluation criteria, also called performance measures, variables, or indicators.

A discipline developed within the operational research and decision engineering areas, and specifically capable of supporting the assessment of alternatives based on multiple criteria is MCDA [143,10,71]. Also defined as Multiple Criteria Decision Making (MCDM) or Multiple Attribute Decision Making (MADM), MCDA is a process that assesses alternatives by identifying the evaluation criteria, eliciting the preferences of the stakeholders and using the preference information to build a preference model that aggregates the multiple criteria evaluations of alternatives. This model permits the comparison of alternatives comprehensively (with, e.g., a ranking, a classification) and leads to a decision recommendation [165,30]. Each MCDA process is composed of different characteristics, which can be defined as features or elements that belong to the MCDA process and serve to identify (parts of) it.

The use of MCDA has increased consistently over the years, due to recognition of the need to address challenges from a multitude of perspectives while dealing with several trade-offs, which calls for multiple criteria assessments [24,94,51,73]. This trend has been particularly marked in complex decision making domains like energy technologies and systems evaluations [169], urban regeneration planning [33], policy analysis [138], health technology management [131], ecosystem services governance [109], sensors placement [13], resilience and sustainability quantification [107,61], energy policy ranking [42], product recovery activities [9], supply chain management [67], waste recycling [66], freight selector [53], housing affordability [122], raw material supply risk assessment [87], water supply systems [149], and polluted land remediation [81].

Due to the exponential growth of MCDA methods in the previous decades [71], an analyst can find it challenging to select the relevant method for the problem under consideration. The consequences of choosing an inadequate approach among the plethora of available methods are substantial, leading to negligence of some critical aspects of the problem, undesired compromises, and ultimately to a recommendation not aligning with actual problem’s characteristics and preferences of the involved stakeholders. Several papers were proposed to discuss the pros and cons of different MCDA methods according to some descriptors of a decision making problem. They included topics such as compensation between criteria, uncertainty management, the meaning of weights, effects of normalization and aggregation methods [31,101,136,137,23,113,73]. These studies can be of help to the analyst in better framing the potentials and limitations of some MCDA methods.

From a complementary, though different perspective, approaches, frameworks, and Decision Support Systems (DSSs) were proposed to lead analysts in the identification of the appropriate method(s) according to the problem under analysis. The software-supported systems became popular because the users requested tools for systematically leading the MCDA method(s) selection challenge with easily updatable systems, as well as a result of the development of computing power and technology [130]. Early proposals of DSSs for MCDA process development and MCDA method(s) selection/recommendation first appeared in the 1980s. The pioneering work of Gershon and Duckstein [64] looked at a limited number (13) of MCDA methods assessed on factors like accepted measurement scales, time for preference provision, and interactivity of the systems. In the following decades, several other authors proposed procedures for the selection of appropriate MCDA methods, e.g., Tecle [159], Ozernoy [130], Hanne [80], Cicek et al. [29], Rowley et al. [142] and Guarini et al. [76]. Approaches based on qualitative characteristics were also proposed, which add a layer of subjectivity to the overall recommendation [25,26,28,148]. All the studies mentioned above have looked at a wide variety of features that require attention and consideration in driving the selection of the MCDA method(s). As far as the criteria are concerned, the meaning of the weights assigned to them, the strategies to deal with interactions among them, and the uncertainty in the measurement scales have been the main focus. From a preference model standpoint, the type of decision recommendation, the functions that can be used to lead towards a decision recommendation, and the level of acceptable compensation between the criteria have also received constant scrutiny in these DSSs. The issue of rank reversal and the possible support of group decisions are other features that have also been accounted for in these systems. The state of the art for MCDA method(s) recommendation is the DSS recently proposed by Wątróbski et al. [177] that covers 56 MCDA methods, looking at nine characteristics of the MCDA problem, including preferences, uncertainty, and desired outcome.

Past studies that developed DSSs for MCDA method(s) selection built their systems on a formal representation of the methods. In other words, they used a taxonomic approach, which is an excellent tool for explicitly representing systematic knowledge and general mapping of some field [63]. A taxonomy is a formal representation of the concepts that constitute a certain domain. In this case, the taxonomy is formed by a set of features that characterize the MCDA methods and the types of decision making challenges they can accommodate.

As shown above, the up to date studies on the MCDA process have reviewed the use of MCDA methods and/or have focused explicitly on proposing approaches and DSSs to select MCDA method(s) according to the type of a decision making problem at hand. However, none has provided a comprehensive overview of the characteristics (i.e., features) that have been or should be considered when leading the MCDA process and selecting the most relevant MCDA method(s). The available literature looked at limited subsets of characteristics, which nevertheless were often entitled or referred to as taxonomies. In particular, Hwang and Yoon [83] accounted for the cardinality of the set of alternatives, as well as the type and salient features of preference information provided by the DM. Furthermore, Ishizaka and Nemery [85] considered the type of approached decision problem and the effort level related to the specification of preference information. On the contrary, Dias et al. [41] distinguished the MCDA methods based on the type of preference model used to synthesize the performances on different criteria as well as the dependency of the comprehensive evaluation of one alternative on other alternatives. Further, Thokala and Duenas [163] referred to the use of weights, the way of measuring the per-criterion performances, incorporating uncertainty analysis, and intuitiveness of the results. Such specifically established taxonomies involve a significant level of subjectivity related to the personal view of the authors on which features are essential or the specific purposes of the assessment related to a particular application domain. In turn, this review aims at discussing the presence and coverage extent for all relevant features. What is more, almost three decades ago, a systematic axiomatic analysis of decision processes and algorithms was advocated by French [59], and this contribution is still missing, as recently confirmed by Wątróbski et al. [177]. Given an extensive literature on the organization of the MCDA process, the time seems to be ripe for mapping this research domain in a comprehensive taxonomic framework, hence forming an appropriate basis for systematic generalization of the MCDA methods.

Driven by the research gaps mentioned above, this paper has three objectives. First, we propose a taxonomy of the MCDA process that enables describing and distinguishing the many methods proposed for MCDA. Its added value derives from the comprehensiveness as well as modularity of the relevant features. The taxonomy provides a general mapping of the MCDA domain, offering a panoramic view of the existing knowledge and the gaps in the state-of-the-art. Second, we advance a clustering of the existing DSSs that have been proposed for recommending MCDA method(s). In this way, we pave the way to a traceable development of systems that can help analysts during their decision support activities. Third, we propose an approach to operationalize the taxonomy for the selection of MCDA methods according to the type of Decision Making Situation (DMS). By formulating a set of intuitive questions referring to the proposed features and showing how decision making can be split into manageable and justifiable steps, we pose the basis for guiding an analyst in choosing the most appropriate methods for a given MCDA problem.

This research is focused on MCDA methods that handle a discrete set of alternatives, where the objective is to comprehensively evaluate them in view of choosing the best one, or ranking them from the best to the worst, or assigning them to predefined and preference ordered decision classes [83,17].

The paper is organized as follows: Section 2 presents the methodology that was used to gather the studies for the review. Section 3 proposes a taxonomy of the MCDA process characteristics. Section 4 describes the clustering of DSSs used to recommend MCDA methods. Section 5 presents a proposal for the application of the taxonomy to lead to the selection of MCDA methods. Section 6 discusses the main findings and concludes by providing some recommendations for future research.

## 2. Methodology

**Fig. 1.**
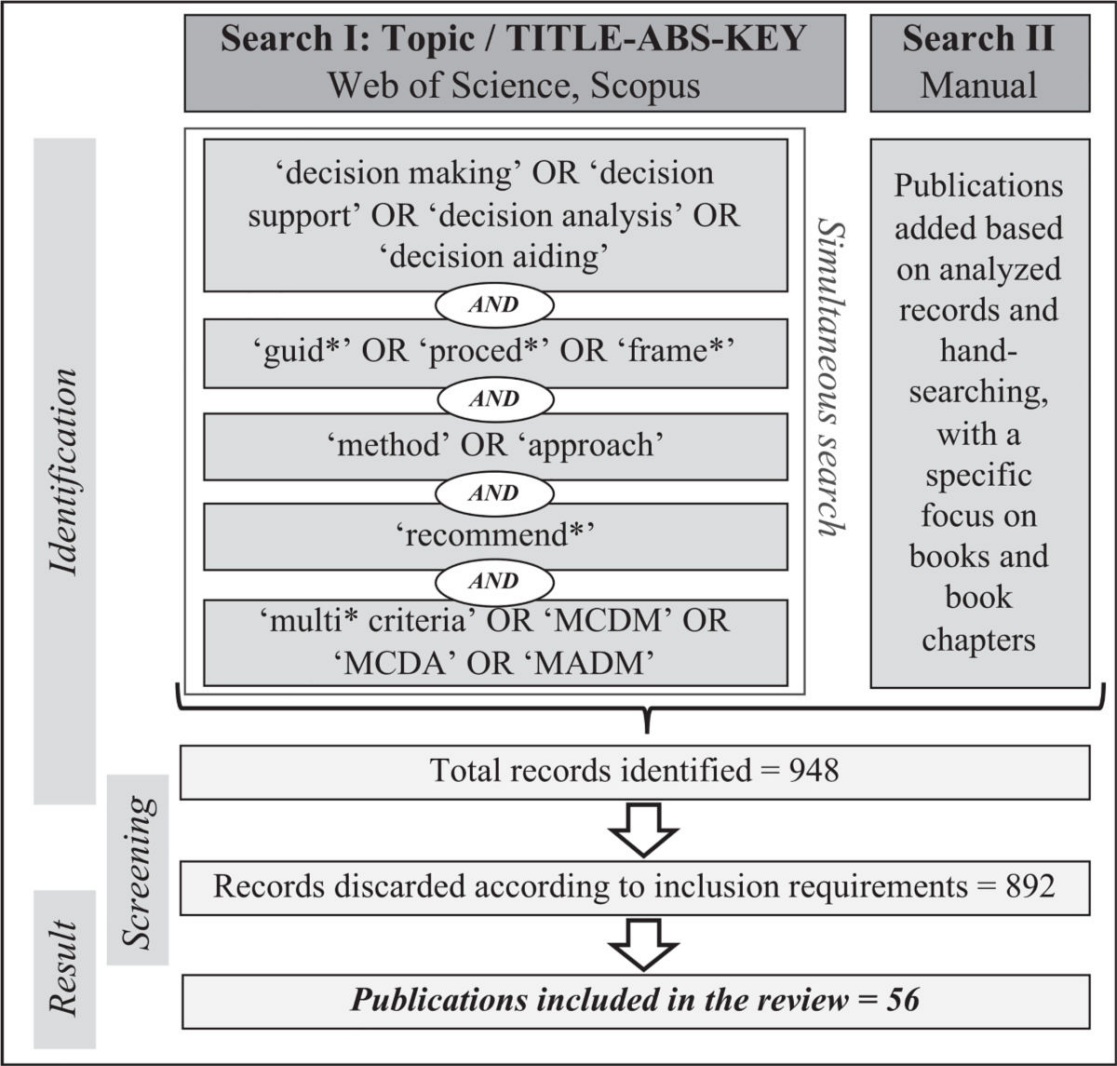
The methodology used to search and select the publications included in the review.

This research explores the existing literature, up to January 2020, on the conduct of the MCDA process, comparison of MCDA methods, and the recommendation of a relevant MCDA method or a subset of methods. The studies were identified in the peer-reviewed literature, more specifically Web of Science and Scopus databases. To find the relevant publications, two searches were conducted. The first one used a combination of keywords in the topic/title, abstract, and keywords within the Web of Science and Scopus database. The main objective of the first search strategy was to select only publications that specifically addressed the challenge of looking at the MCDA process, its components, and its methods working strategy with a “holistic view”. In other words, the Boolean “AND” was used to make ensure selecting literature that specifically looked at supporting the analysts in conducting the MCDA process, traceably framing an MCDA challenge, and recommending a method. Furthermore, the second search was a manual one, that was less restrictive and included the combinations of words like “MCDA” and “framework”, or “recommend” and “MCDA”, or “decision support” and “recommend” and “MCDA” or “MCDM” or “MADM”. Fig. 1 shows the overall search strategy and results. The search reported 948 publications, which were screened and included in the analysed dataset if they provided an overview and/or a set of steps to lead the MCDA process and also satisfied one or more of these inclusion requirements:

- Provide guidelines, approaches and/or a set of steps to choose an MCDA method or a subset of methods;
- Discuss the strengths and weaknesses of MCDA methods;
- Discuss the suitability of certain MCDA methods to certain problem types;
- Provide reviews or frameworks of the available strategies adopted to perform assessment of alternatives based on the MCDA process.

Publications that exclusively focused on theoretical considerations on MCDA, individual MCDA methods and/or simply described a case study using MCDA were excluded. Furthermore, the factual reporting of the use of MCDA methods according to certain features (e.g., year of publication, journal, authors’ nationality) was not used to populate the taxonomy.

56 publications of the 948 met the inclusion criteria, and they were clustered in two main groups (see Appendix A in the Electronic Supplementary Information (ESI) for a full list of studies):

1. Those supporting (part of) the MCDA process and comparing MCDA methods ( *N* = 33);
2. Those presenting DSSs recommending a specific MCDA method or a subset of MCDA methods ( *N* = 23).

These publications contained a vast amount of information, which was condensed in taxonomy in order to provide a consistent overview of the features of the MCDA process. A taxonomy can be very useful in this regard as it is specifically tailored to help in (i) development of a comprehensive perspective on a domain, (ii) making the components (attributes) of a domain explicit, (iii) understanding the links between the various attributes, and (iv) identifying potential operational or knowledge gaps [50]. For these reasons, this paper proposes a taxonomy of the characteristics of the MCDA process. The taxonomy has been developed from the MCDA characteristics (i.e., elements) presented in the studies included in the review and iteratively assessed with respect to its coherence and comprehensiveness based on the relevant literature on the MCDA process [16,123,18,14,71,45].

## 3. A taxonomy of the MCDA process characteristics

**Fig. 2.**
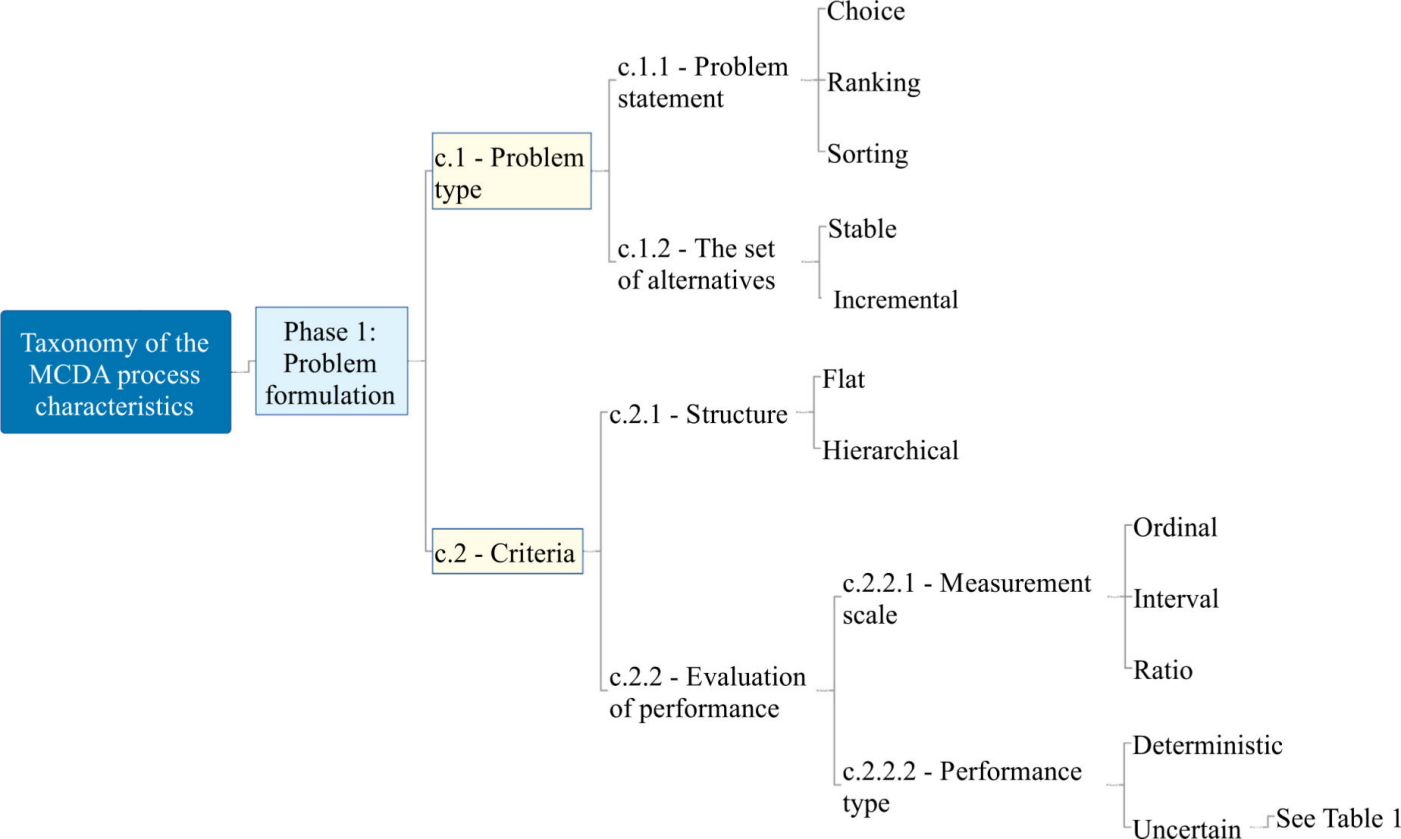
Taxonomy of the MCDA process characteristics, Phase 1 - Problem formulation.

**Fig. 3.**
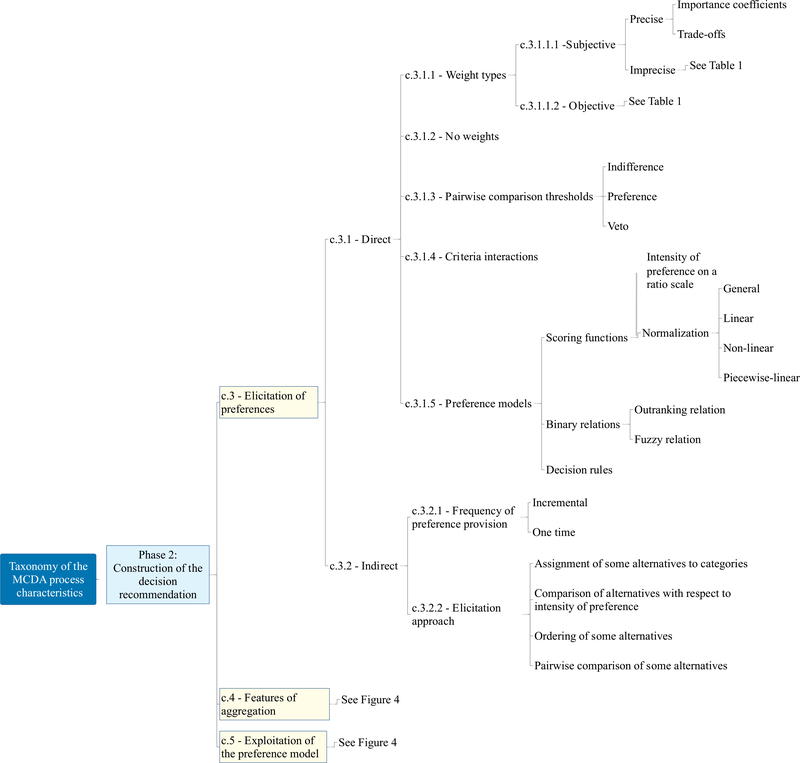
Taxonomy of the MCDA process characteristics, Phase 2 - Construction of the decision recommendation: Elicitation of preferences.

**Fig. 4.**
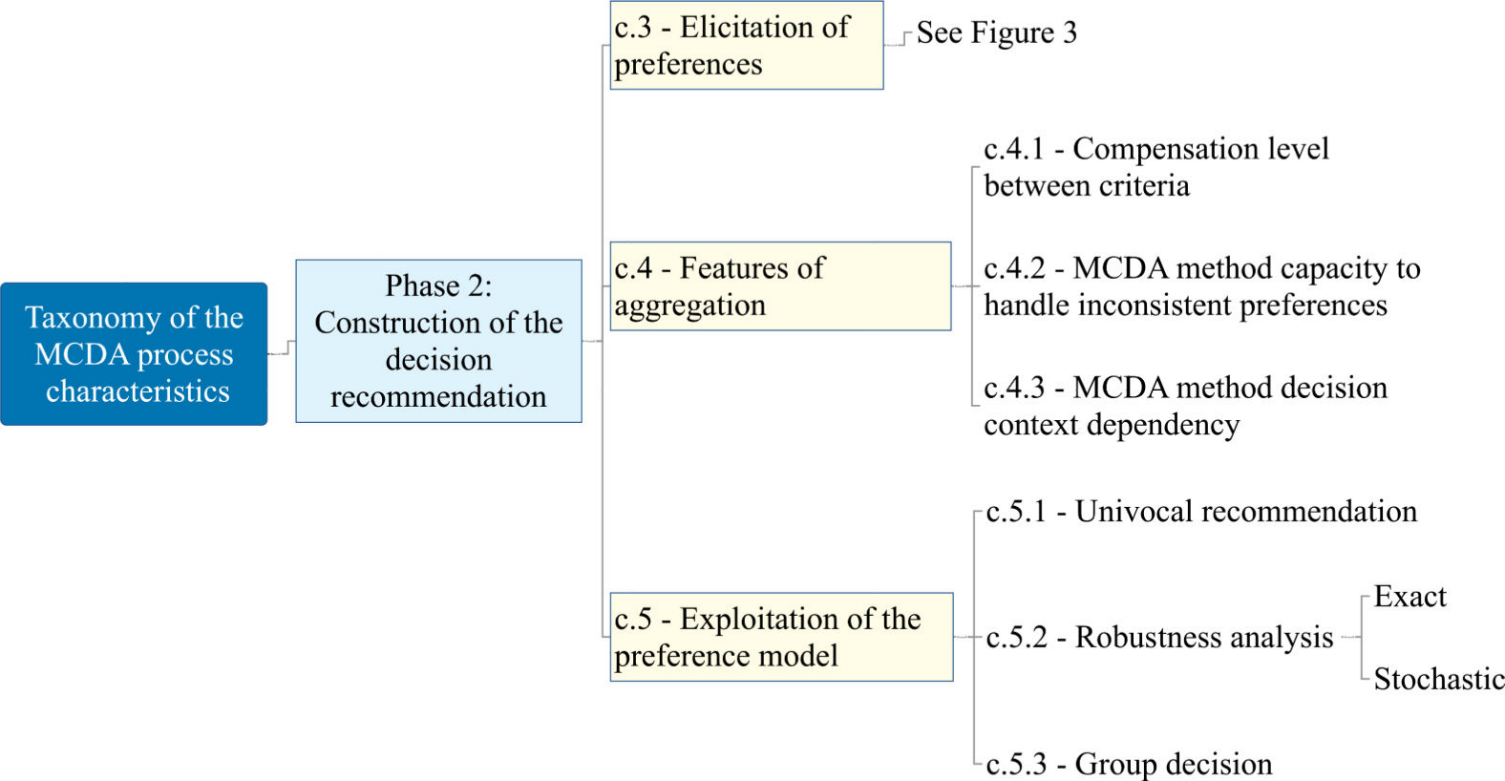
Taxonomy of the MCDA process characteristics, Phase 2 - Construction of the decision recommendation: Features of aggregation and exploitation of the preference model.

**Fig. 5.**
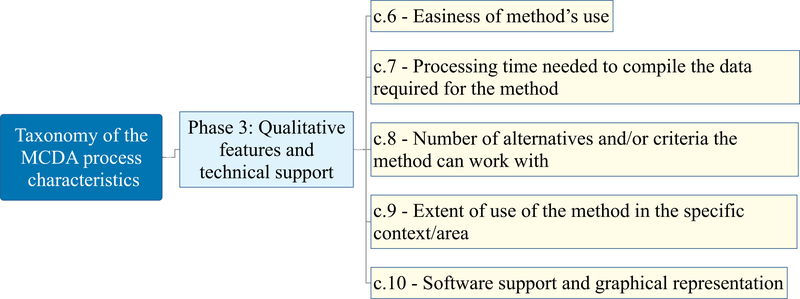
Taxonomy of the MCDA process characteristics, Phase 3 - Qualitative featuress and technical support.

The taxonomy of the MCDA process characteristics has a hierarchical structure; it is composed of three main phases and includes ten main characteristics. Its structure (with each characteristic and sub-characteristic labelled using a “c.” and “c.x.” nomenclature, respectively) is available in Figs. 2, 3, 4, and 5 and the complete taxonomy is summarized in Table 1 with a breakdown of frequency and share of each characteristic with respect to the 56 reviewed studies. Appendix A in the ESI provides a complete list of the reviewed studies with the indication of whether each characteristic of the taxonomy was considered or not by each study. Appendix B in the ESI gives a tabular description of the taxonomy characteristics.

Phase 1 is labelled *problem formulation,* and it is summarized in Fig. 2. It focuses on the initial building blocks of the MCDA process [12], where the structure of the problem is defined by identifying the set of alternatives, the desired type of decision recommendation, and the structure of the criteria, as well as the performance of the alternatives. More specifically, phase 1 includes the first two characteristics, and it deals with how the problem is framed by looking at the type of decision making challenge under consideration (c.1) and the criteria used to assess the alternatives (c.2). This includes two main clusters. The first is problem type, distinguishing the type of recommendation desired by the model (i.e., choice, ranking, sorting) (c.1.1) and its possible dynamic character (i.e., the stability of the set of alternatives) (c.1.2). The second looks at the structure of the criteria, which can be flat or hierarchical (c.2.1), and their evaluation of performance (c.2.2), on such scales as ordinal, interval, and ratio ones (c.2.2.1). Another distinction included in the evaluation of performance is that it can either be deterministic, with precise values, or non-deterministic, with uncertain values (c.2.2.2).

Phase 2 is named the *construction of the decision recommendation,* and is framed in Figs. 3 and 4. It follows the formulation of the problem by studying how the preferences of the Decision Maker(s) (DM(s)) are elicited (c.3), what requirements are placed by the user while considering some methodological features of the aggregation method (c.4), and how the preference model can be exploited to provide the decision recommendation (c.5). The elicitation of preferences (c.3), which is fully shown in Fig. 3, refers to the type of preference information that will be used to develop the model, either direct or indirect. The features of aggregation (c.4 in Fig. 4) include the topics of compensation between the criteria performance (c.4.1), the capacity of the MCDA methods to deal with inconsistent preferences (c.4.2), and the dependency of the recommendation on the decision context (c.4.3). The latter refers to the susceptibility of the user/DM to the possible rank reversal that can occur when new options are added to the pool of considered alternatives. Lastly, the strategies for exploiting the preference model (c.5 in Fig. 4) look at how a such model that is shaped based on the preferences of the DMs/stakeholders can be used to reach the decision recommendation, which completes phase 2.

Phase 3 is classed *qualitative features and technical support* (c.6–10), and it is presented in Fig. 5. This phase includes characteristics that the user might want to take into account when choosing the MCDA method that fits with his/her decision making challenge. The characteristics in this phase do not objectively steer the recommendation of MCDA method(s), as it should be in the case of phase 1 and 2. These are rather related to the mathematical literacy of the analyst and/or DMs/stakeholders (c.6), time constraints and resources availability (c.7), technical capability and extent of use of the MCDA method (c.8 and c.9), and lastly software implementation of the MCDA method(s) and results visualization capacities (c.10).

Each characteristic is now presented and discussed in detail in the following sub-sections from 3.1 to 3.8.

### 3.1. Problem type (c.1, phase 1)

This characteristic (upper part of Fig. 2) considers the type of recommendation desired by the user (c.1.1), also referred to as problem statement in MCDA terminology. The most common problem statement encountered is ranking (75% out of *N* = 56 studies), with sorting and choice still included, but at a lower ( ∼45%) share (see Table 1). Ranking consists in imposing a preference relation on the set of alternatives, i.e., listing them from the best to the worst. Sorting (sometimes also referred to as ordinal classification) means to assign the alternatives to pre-defined preference-ordered classes (sometimes called categories). Choice refers to the selection of a subset of the best alternative(s) [145]. Each categorization can, however, be expanded by looking at the desired type of order of the set of alternatives (partial, complete). The possibility of using incomparability relations allows deriving partial orders of alternatives. On the one side, this has the advantage of highlighting alternatives that are considerably dissimilar to each other. On the other side, it can confound the DM who might desire a univocal complete ordering. Another important consideration is that the final recommendation can be based on scores of alternatives or on binary relations. The former (i.e., scoring) can result in a cardinal type of recommendation, where the distance between each alternative is meaningful in quantitative terms. The latter form of recommendation (i.e., binary relations) leads to an ordinal recommendation, where only the position of the alternatives is meaningful.

Some more refined problem statements have also been proposed in the literature, like design, description, portfolio, nominal classification, or clustering problematics [3]. However, they have not been found in any of the studies in this review, which indicates the three selected statements proposed are sufficient to represent the type of problems normally encountered by the analysts.

This characteristic also includes the possible dynamic character of the problem in relation to variability in the number of alternatives (c.1.2). Evolving sets of alternatives can emerge due to the unstable character of the decision context. It has been stated that, when the set of alternatives is evolving, relative comparisons of alternatives become less stable, and consequently models that focus on the independent assessment of each alternative should be preferred (e.g., sorting ones involving a comparison of alternatives with an externally defined reference [168]).

### 3.2. Criteria (c.2, phase 1)

The characteristic that focuses on the criteria themselves (lower portion of Fig. 2) distinguishes between their structure (c.2.1), their type of measurement scale (c.2.2.1), and the type of performance used as input data (c.2.2.2).

#### 3.2.1. Criteria structure (c.2.1)

The complexity of multiple criteria-based projects often requires analysts to structure the criteria in a hierarchical form. This enhances the manageability of the data, which can be handled with small groups of criteria, and also allows performing a conceptual clustering in main categories [21,110]. Some examples of research areas where criteria clustering is common are sustainability [162], resilience [62], urban regeneration [15], water resources management [79], and technology comparison [164,169]. In these domains, a hierarchical decomposition of problems involving a large number of heterogeneous criteria (e.g., economic, social, and environmental) proved to be useful from the structural and functional viewpoints. This strategy of problem structuring has been confirmed by this review, which shows that 32% of the studies consider the possible use of criteria hierarchies, as summarized in Table 1.

#### 3.2.2. Measurement scale (c.2.2.1)

It is confirmed that most (70%, see also Table 1) of the studies reviewed discuss the use of different measurement scales of evaluation criteria and the capability of MCDA methods to manage them. Ordinal, interval, and ratio scales are presented as the three measurement options. Interval and ratio mostly belong to the physical-technical application areas, while the ordinal one is very common in the socioeconomic as well as sustainability/resilience assessment domains [128,61]. The interval and ratio scales are often called cardinal. A more general distinction regarding measurement scale use is between qualitative and quantitative [114,115,175]. In the latter case, the authors stress the need of being aware of the fact that MCDA methods exploit information differently, some allowing one to account for the distance between the performance by using the quantitative nature of the input (e.g., MAVT), while some others do not, and only use their qualitative nature (e.g., ELECTRE II).

#### 3.2.3. Type of performance (c.2.2.2)

An important distinction that appears in the problem formulation phase is the type of performance of the alternatives, which can be divided into deterministic and uncertain. Due to the difficulty in obtaining precise performance, possibly due to the costs involved, and also the desire to explore the stability of the results when accounting for the variability of the input data, it was found that uncertain performance is a recurrent and diversified topic. Fuzzy and probabilistic approaches appear to be the most common ones to deal with the uncertain performance of the alternatives [43,132], which might be due to the rigor, flexibility and powerful communication potentials of both approaches. The probabilistic approach has been mostly characterized by the use of stochastic methods, with probability distributions of different shapes assigned to the possible performance data, possibly based on empirical uncertainty characterization methods. The fuzzy approach allows dealing with the vagueness of information, by specifying a sort of soft transition from one qualitative performance level to another (e.g., from ‘poor’ to ‘fair’), without an abrupt change of the degree of possibility. Other techniques commonly used to model uncertain performances are evidential reasoning and gray numbers. In the former approach, each performance is represented using a belief structure defined by a distributed assessment. In contrast, the latter approach incorporates performances specified by means of clear boundaries without indicating an exact position of the performance within these boundaries. An interesting proposal that advances the state of the art with respect to the use of uncertainty modeling techniques integrated with MCDA method, is the decision support framework presented by Pelissari et al. [132]. This framework helps with choosing the uncertainty modeling technique according to the type of data uncertainty, whether related to ambiguity, stochasticity, or partial information.

### 3.3. Elicitation of preferences (c.3, phase 2)

Preferences of the DMs/stakeholders can be used to shape the structure of the model, and they can be provided with direct (c.3.1) (left branch of Fig. 3) and indirect (c.3.2) methods (right branch of Fig. 3). These are not mutually exclusive, as some methods can accept preferences which can be direct as well as indirect.

#### 3.3.1. Direct preferences (c.3.1)

Direct preferences are directly provided by the user/DM, and they include:

- Weight types (c.3.1.1): used to express the relative importance of the criteria. These can be precise and imprecise, with precise subsequently divided into trade-offs, i.e., substitution rates, and importance coefficients, i.e., voting power. While for the imprecise, no precise values are defined, and they are driven by constraints of different types (e.g., ordering of the criteria, pairwise comparisons). There can also be cases where weights are not used (c.3.1.2);
- Pairwise comparison thresholds (c.3.1.3): characterize the preference sensitivity of the DM(s) when comparing two alternatives. The basic three types include: indifference, preference, and veto;
- Interactions (c.3.1.4): consider the links, dependencies, and trends between the criteria;
- Preference models (c.3.1.5): distinguish the main clusters of MCDA modeling philosophies, being (i) scoring functions, (ii) binary relations, and iii) decision rules.

There can be cases where these preferences are not known, such as missing weights or uncertain thresholds and interactions. In such cases, they can be handled with approaches like identification of the non-dominated fronts, which exploits only the performance of alternatives [40], or Stochastic Multi-criteria Acceptability Analysis (SMAA) where the missing information is modeled as a distribution that can cover the whole spectrum of possible values [99]. It must be stressed that one issue of concern is the choice of the shape of such distributions, which is usually an implicit uniform one. Further attention could be devoted to the strategies that can lead to a relevant definition of the shape of these distributions while matching the DM’s value system.

In the reviewed publications, it was found that direct techniques to elicit weights, thresholds, and interactions are mostly looked at. These approaches are useful for the learning experience of the people involved in the process, though they have the drawback of being (sometimes very) cognitively demanding and also time-consuming [52]. Some examples are the direct construction of value functions, the definition of preference, indifference, and veto thresholds, or the elicitation of weights as trade-offs [148].

##### 3.3.1.1. Weighting (c.3.1.1).

A key phase of the MCDA process is the weighting of the criteria, the first to appear in phase 2 of the taxonomy, as shown in Fig. 3). The objective here is to assign the criteria certain relative importance, which can be defined either by the experts/DMs (c.3.1.1.1) or according to the data structure and correlations (c.3.1.1.2).

###### 3.3.1.1.1. Subjective weighting (c.3.1.1.1).

This review shows two main distinctions for the weights that can be subjectively provided by people involved in the MCDA process. The first one is between the techniques used to derive precise weights from those used to obtain imprecise weights. The second division is within the precise weights’ category, where there are two sub-categories of weights with a very different conceptual meaning, trade-offs on one side and importance coefficients on the other. Even if only a minority of publications discusses this difference of meaning between the precise weights, it is a fundamental distinction, since weights as trade-offs are necessary for MCDA methods that accept full and partial compensation and operate with a score-oriented aggregation, whereas weights as importance coefficients can be used with outranking approaches [125]. Furthermore, weights as importance coefficients are particularly suited when highly different dimensions are considered, such as physical, economic, and social data, for which it is particularly difficult if not impossible to elicit tradeoffs [128]. It is also important to note that weights as importance coefficients refer to the criterion itself and not to its quantification (i.e., measurement scale), while trade-offs are directly linked and dependent on the criteria scale, which must be quantitative [126].

The use of uncertain weights has received increasing attention because it allows for deriving a distribution of outcomes (e.g., ranking of the alternative), that can be used to test the robustness of the evaluation [132]. This has the added advantage of tackling the issue of representative weights (i.e., using a single set of weights), which has been criticized as it implies these weights are valid for a whole population, which is not true in most cases [73]. Uncertain, as well as multiple sets of weights, allow developing an evaluation that considers a multitude of perspectives and provides a sense of confidence about the stability of the results, which the single input approaches cannot accommodate [106]. This topic is directly linked with the exploitation of the model, which has been mostly looked at from a stochastic robustness viewpoint, by using the distribution of the inputs to assess whether there is any trend and/or stability in the results (e.g., with Monte Carlo Simulations) [21,43]. In addition, uncertain preference information is less complex to elicit and more stable compared to the certain one [116]. This constitutes a notable advantage when considering the limited time that DMs have to interact with the analysts.

###### 3.3.1.1.2. Objective weighting (c.3.1.1.2).

Another weighting avenue that has been less discussed in the literature (below 10%) is the use of data-driven weights, which are defined according to the variability of the criteria and relationships between the criteria [112,73]. Some examples used to assign these weights are correlation analysis, factor analysis, and principal component analysis [128]. They have been criticized because of the lack of influence of the DM on this crucial step of the MCDA process, whose primary aim is to incorporate their preferences in the decision making process [73]. It is nonetheless important to be aware of the fact that the structure of the data has an effect on the influence that each indicator has on the index when scoring functions are used as preference models. Consequently, an informed choice of the weights should also be combined with a sound statistical analysis of the dataset [11].

###### 3.3.1.2. Thresholds (c.3.1.3).

Around one-third of the studies looked at the pairwise comparison thresholds, which can be used for handling uncertainty in the measurement of performance, as well as to deal with the hesitation of the DM [135,21,146]. The indifference thresholds account for the maximum difference that makes two alternatives indifferent. The preference thresholds consider the minimum difference in performance that leads to a full preference of one alternative over the other. In some MCDA methods, like ELECTRE, an additional threshold called “veto” can be used to enforce fully non-compensatory modeling. This threshold guarantees that when an alternative performs worse than another one by at least the veto value on even a single criterion, then the former cannot outrank (i.e., be considered as good as) the latter, irrespective of its comparative performance on the remaining criteria [144,135]. For the sake of completeness, in ELECTRE, there are also other thresholds that were not mentioned in the review studies, being the pre-veto (discordance), reinforced-preference and counter-veto [120,147], all used to refine the pairwise comparison preferences that the DMs might have.

##### 3.3.1.3. Interactions (c.3.1.4).

Only 11% of the studies considered the topic of interactions between the criteria (see Table 1). A common assumption in MCDA model development is the preferential independence between the criteria. In other words, criteria are assumed not to interact. Essentially, preferential independence implies that the contribution of the performance on a given criterion into the alternative’s comprehensive evaluation is not affected by its performances on the remaining criteria. This simplification allowed developing simpler, usually additive models.

Nonetheless, real life is more complex, and interactions between criteria are rather common, which justifies the development of models that can account for them. Criteria can interact positively, meaning that their importance considered together is higher than the sum of the importance of these criteria considered separately. Conversely, criteria are negatively interacting when the importance of criteria considered together is lower than the sum of the importance of these criteria considered separately. An example of positive interactive criteria is the maximum speed and the price of a car. Usually, a car that runs at a high maximum speed is also expensive. Cars which have high maximum speed and low price are thus particularly appreciated. Hence, the importance assigned to the combination of maximum high speed and price is higher than the sum of these two criteria taken separately. An example of negative interaction can be two criteria whose calculation is driven by similar input. Consequently, when one of these criteria has a good performance, the same happens for the other, and vice-versa [4]. Advanced MCDA models handle interactions of yet different types (see, e.g., the antagonistic effect in ELECTRE) or concerning subsets of more than two criteria.

##### 3.3.1.4. Preference models (c.3.1.5).

There appears to be some widespread agreement on the clustering of aggregation algorithms used by MCDA methods in three main groups. As summarized in the central part of Fig. 3 [160,176,70,73], they include (i) scoring functions, (ii) binary relations, and (iii) decision rules. Most of the methods in the first group implement a common procedure called normalization, which brings all the measurement scales of the criteria on the same scale and then applies some form of aggregation to derive a comprehensive score that can be used to rank, classify, or choose the most preferred alternative [128,84]. A common distinction presented in this group is between value- or utility-based methods [108]. Another method that, together with its extensions, belongs to this category is the Analytical Hierarchy Process (AHP) [151]. AHP finds priorities of the alternatives using pairwise comparisons of alternatives provided by the DM with respect to each criterion. The preference intensity of these pairwise comparisons is expressed on a ratio scale (usually between 1 and 9). Yet, another sub-group within this category is formed by the distance-based methods with TOPSIS [83] being the main representative. These approaches derive a score of each alternative based on its distance to one or more points in the multi-dimensional performance space. The methods in the second group perform pairwise comparisons of the alternatives leading to an outranking matrix, which represents a directed graph where alternatives are in nodes and outranking relations are on arcs, and the quality of each alternative depends on its relations with all the other alternatives. This matrix is then exploited using different algorithms (e.g., net flow score procedures or approaches for identifying a graph kernel) to develop the final recommendation. The third group is the decision rules one, based on logical “if…, then…” statements that represent scenarios of a causal relationship between the performance of alternatives on a subset of criteria and a comprehensive judgment [75]. There are however some studies that prefer to propose a general category named as “others” after the first two presented earlier [78,154,142], where elementary MCDA methods and/or combinations of MCDA methods are included, like lexicographic method, maximin method, and (fuzzy) conjunctive/disjunctive method.

A unique presentation of method categorization and potentials in terms of data treatment has been given by Moffett and Sarkar [114], who describe the MCDA methods according to the way they exploit preference information. The authors start by describing those methods that use the information without any weights, like the non-dominated set computation and maximin, moving then to those that use qualitative weights, and finally to those that use quantitative weights. The same incremental scheme with respect to the weighting (i.e., no - > qualitative - > quantitative weights) is then applied to the methods that use quantitative data. This approach provides a useful educational strategy to distinguish methods according to their way of dealing with preference information on the criteria (i.e., weights) and the way the input data is used (i.e., qualitative or quantitative).

MCDA methods can also be described in terms of axiomatic foundations, which allows defining the preference model from the viewpoint of the weakest mathematical assumptions that have to be adopted in order to construct a given preference model. This aspect has been stressed within a decision-theoretic approach by the studies of Roy and Słowiński [148], Munda [124], and Bouyssou et al. [17] only. Examples of such axiomatic analyses of preference models of all three types have been performed by Słowiński et al. [156] and Greco et al. [74]. Indeed, when selecting an MCDA method, it is important to make sure that such an axiomatic characterization of the method is understood by the analyst and accepted by the DM. This aspect is, however, a significant challenge, given that many DMs (and sometimes analysts too) do not find themselves much at ease with axiomatic mathematical formulations [17] nor competent enough to answer formal questions related to the axiomatic verification. One partial solution could be to “test” the axioms in real practice, by showing the DM a few pairs of alternatives and checking whether his/her preferences fit with the axioms [17].

As far as the first family of methods is concerned (i.e., score-oriented), this review shows that popular normalization strategies, which are used to harmonize the measurement scales of the criteria, include approaches that (i) are only driven by the ordinal nature of the data (defined as “general approaches”), ^1^ while the others actually account for the cardinal differences in the performance including (ii) linear, (iii) piecewise linear and (iv) non-linear transformations [128]. The “general” group includes methods such as rank, percentile rank, and categorical, which use the position of the alternative in the ranking [128]. The linear cluster refers to, e.g., the min-max, target, and distance to a reference approach [23], while the piecewise linear is the characteristic method for the development of value functions [55]. The non-linear category includes the approaches that do not fit into any of the previous ones, with two examples being the standardized [128] and logistic [22].

#### 3.3.2. Indirect preferences (c.3.2)

Methods that work with indirect preferences differ from those that use direct ones in that they elicit the model parameters from local or holistic judgments of the experts/DMs. These preferences can either be provided one single time, resulting in the final model as soon as the input is received, or in an incremental manner (c.3.2.1), so that the experts/DMs give their judgments gradually and the model is refined each time their new input is accounted for. The right side of Fig. 3 depicts this distinction. The preferences of the DM (see c.3.2.2) can include the assignment of some alternatives to decision classes (e.g., alternative 1 can be assigned to class good), pairwise comparisons of some alternatives (e.g., alternative 1 is preferred to alternative 2), or ordering of some alternatives (e.g., alternative 1 is better than 2, which is better than alternative 3). Weights, thresholds, and shapes of the model functions can then be derived from these indirect preferences and used to build the decision recommendation.

### 3.4. Features of aggregation (c.4, phase 2)

This characteristic accounts for key considerations about the implication of the choice of the MCDA method with respect to the level of compensation (c.4.1), the capacity of the MCDA methods to deal with inconsistent preferences (c.4.2), and the dependency of the recommendation on the decision context (c.4.3). It is still part of phase 2 of the taxonomy, and it is presented in the middle of Fig. 4.

#### 3.4.1. Compensation (c.4.1)

A topic that has attracted wide attention is compensation ( ~60% of the studies as presented in Table 1), which refers to the admissible trade-offs between criteria performance and can range from a full to a null level, with a wide spectrum of possibility in between. The compensation level can be driven differently according to the aggregation algorithm used by the MCDA method [78,126]. As far as score-oriented aggregation methods are concerned, their compensation can range from a full level with the additive weighted mean to a low level, e.g., with the harmonic mean [101]. Combinations of these means can be created to tune the level of compensation according to the desires of the DM [101]. In the case of outranking methods, they have been specifically developed to handle low and null degrees of compensation, which is primarily justified by the pairwise-based comparison of alternatives to derive the decision recommendation [126,31]. As far as the decision rules are concerned, they are inherently non-compensatory due to the nature of their modeling, since it is necessary to reach the conditions of the rules to trigger any recommendation [158].

#### 3.4.2. Inconsistent preferences (c.4.2)

As far as the capability to manage inconsistent preferences is concerned, only one of the selected studies mentioned it [148]. This is an important feature to account for, especially when MCDA methods use indirect preferences. Inconsistencies appear when the preferences are not compatible with the assumed model, e.g., due to the underlying strict axioms (e.g., additivity, monotonicity, or preferential independence) or the DM’s judgments are conflicting (e.g., when the DM assigns two alternatives with identical performances on all criteria to different decision classes, or when the DM prefers a pairwise comparison for an alternative that is dominated by the other alternative with respect to all considered criteria). The latter can be due to the error made by the DM in his/her statements or some criteria not being included in the considered family of criteria, though still influencing the DM’s decision.

Inconsistent preferences can be handled using the rough set concept, which allows structuring the input preference information prior to the induction of a decision model in the form of decision rules. In consequence, decision rules based on consistent preference information show certain relationships between the evaluation of alternatives on considered criteria and DM’s decision, while decision rules based on the inconsistent preference information show ambiguous relationships. This methodology has been implemented in the Dominance-based Rough Set Approach (DRSA), which develops decision recommendations in terms of decision rules [75]. The rules that constitute the decision model and are based on inconsistent preference information are highlighted with a consistency ratio, informing the DM that the recommendation provided by them is based on partially consistent preference information. Alternatively, as noted by Ghaderi and Kadziński [65], in decision aid practices where an interaction with the DM is possible and desirable, the DM can be asked to revise some judgments to restore the consistency or to pursue the MCDA process while accepting some level of inconsistency. In the former case, the DM may be exhibited with the minimal subsets of judgments to be dropped. In contrast, in the latter case – she may be offered the model minimizing a total error related to the reconstruction of the DM’s judgments by an assumed preference model.

#### 3.4.3. Decision context dependency - Rank reversal (c.4.3)

The addition or deletion of alternatives can lead to a change in the problem structure, which can have implications on the decision recommendation. The most common phenomenon is the rank reversal, which consists of a possible inversion of the ranking of the alternatives when a new (or more than one) alternative is added to (or deleted from) the set [49]. It has been observed in several MCDA methods, including AHP, weighted sum with internal normalization, and TOPSIS [172]. This review found that rank reversal has been considered in ∼20% of the studies (see Table 1). This issue has received particular attention for the AHP method, as summarized in the review article by Maleki and Zahir [105]. In consequence, there were proposals of versions of AHP that can avoid this phenomenon [171,1].

As far as ELECTRE methods are concerned, only Wang and Triantaphyllou [170] and Figueira and Roy [57] studied the topic and provided different interpretations. The former states that it is due to the exploitation procedure of the outranking relation, implying that it is the major issue in the method. The latter justified the rank reversal as a consequence of the pairwise comparison expressed by the outranking relation and argued that it is not compulsory to avoid rank reversal when the preference relation is assumed to be non-transitive. Thus, in this case, the rank reversal can be accepted and should not be seen as a limitation. This reasoning applies to all methods using outranking relations, among which ELECTRE and PROMETHEE. In the case of PROMETHEE, it has been shown that significant rank changes can only happen when a large number of alternatives are added to the set [141].

However, it must be noted that changing the alternatives in the set also leads to a change in the problem setting and the comparisons between the alternatives. This has been presented as an acceptable justification for the change in the rankings by methods that work with pairwise comparisons [57].

### 3.5. Exploitation of the preference model (c.5, phase 2)

The last part of phase 2 of the taxonomy, shown in the right side of Fig. 4, considers the stage of the MCDA process when performance on all considered criteria, as well as the preferences of the DM are defined, and the preference model can be built and exploited to derive the final decision recommendation. The exploitation can suggest to aggregate the provided information in a single score to rank the alternatives or consider the binary relations between the alternatives to assign them to preference-ordered classes. In the event of univocal inputs, a unique solution can be obtained by means of a single preference model (c.5.1). This is the ubiquitous considered strategy, with all the selected literature mentioning or implicitly referring to it as a form of final recommendation of the MCDA method (see also c.5.1 in Table 1). Alternatively, when there is uncertainty with respect to the performance of alternatives and/or representation of the DM’s preferences by an assumed preference model, the exploitation of the latter one can be conducted from a robustness perspective (c.5.2). Two approaches have been found in the literature to conduct such exploitation. The first is the exact mode, where all instances of the preference model compatible with the uncertain performance and/or DM’s preferences are exploited [38]. Depending on the characteristics of performance and preferences, it can be implemented either by analyzing the recommendation obtained with a finite number of all compatible model instances or – in case these cannot be listed explicitly – by using mathematical programming techniques which exploit infinitely many compatible model instances delimited by a set of constraints. The other is the stochastic mode, where some distribution of the performance/preferences is assumed, and a subset of all compatible model instances is sampled from the distribution and the outcomes obtained with their use are summarized to verify the stability of different parts of recommendation [98,92,35]. The most popular tools used to implement such a stochastic mode are Monte Carlo methods.

Let us emphasize the difference between robustness and sensitivity analyses. The ability to conduct the former can be considered as an inherent feature of a particular MCDA method because it consists of accounting for the uncertainties observed in the actual decision situations already at the stage of developing the model and deriving a recommendation for a particular problem [168]. Specifically, a conclusion is considered to be robust if it is valid for all or most plausible sets of performance or all values of the preference model parameters compatible with the preference information [44].

In other words, robustness analysis assesses how variable the outcome of the model is while accounting for all the uncertainties that are included in the model. On the contrary, sensitivity analysis consists in verifying if and how the recommendation already derived with a particular method would be affected under alternative assumptions on the alternatives’ performance and/or DM’s preferences. More specifically, sensitivity analysis looks at which sources of uncertainty are more influential on the final outcome, such as the weights, normalization methods, thresholds, or aggregation functions [128]. The process of recalculating outcomes in this way can be coupled with any MCDA method. Thus, the ability to conduct sensitivity analysis cannot be perceived as a feature driving the choice of a particular MCDA method.

Another strategy for exploiting the model is when the preferences of a group (c.5.3) rather than an individual are modeled to reach a group decision recommendation. Such methods can be distinguished at different levels [90]. Specifically, they can be divided with respect to the level on which individual viewpoints are aggregated. When aggregating the DMs’ preferences at the input level, a compromise recommendation is constructed for all DMs considered jointly. When the aggregation is conducted at the output level, one identifies the spaces of consensus and disagreement with respect to the results attained individually for different DMs. Furthermore, the group decision making method may assume that all DMs play the same role in the committee or account for the importance degrees of the involved DMs, hence differentiating their roles and impacts on the final decision. Finally, group decision methods can also be distinguished in terms of the number of stakeholders whose opinions can be handled [100]. Depending on the characteristic of the underlying participatory process, the number of stakeholders can be low or high Guarini et al. [77]. In the former case, the method needs to consider the preferences of a limited number of DMs or experts. In the latter case, the number of stakeholders can be up to hundreds or thousands, possibly being organized in different interest groups having their own motives and preference systems. Such problems often appear, e.g., in public environmental problems [100] or patient benefit-risk analysis [161].

### 3.6. Qualitative characteristics (c.6–9, phase 3)

Phase 3 of the taxonomy mostly includes qualitative features, as displayed in Fig. 5. This review has found that up to 36% (see phase 3 of the taxonomy in Table 1) of the included studies consider characteristics that are not method-specific, but rather subjective and driven by the type of analyst and also the DMs/stakeholders involved. Due to the different background and mathematical competency of the stakeholders involved in the decision making process, the main qualitative consideration that several authors mention when selecting an MCDA method is the understandability of the method (c.6). This can also be seen as the easiness of use, referring to the time required for interaction with the DM to explain the method and/or obtain preference information, and the level of input required from the stakeholders [135,148,31,56]. This characteristic can also be affected by the simplicity of structuring sensitivity analysis, who’s complexity can vary according to the type of preference model [56]. For example, if MAVT is used, this can involve changing the shape of the value functions. If PROMETHEE is selected, different preference functions and thresholds can be tested.

Processing time and effort needed to compile the data required for the method (c.7) are other important characteristics. This can vary considerably according to the selected method. For MAVT, it includes weights as trade-offs and value functions. For ELECTRE methods, it can involve weights as importance coefficients and comparison preference and majority thresholds. In DRSA models, it includes some comprehensive judgments on a set of reference alternatives. The complexity varies also according to the number and type of criteria, which is accounted for by the next characteristic, looking at the number of alternatives and criteria (c.8) the method can deal with [129,159,25,112,76]. Due to the lack of deterministic analysis on these capabilities for most of the methods, it is common to see that this criterion scale is qualitative (e.g., high, medium, low).

Another subjective consideration added to this set is the extent of use of the method in the specific context/area (c.9), with the assumption that the more a method is used in a certain area, the more it is seen as the correct method [115]. This is a risky assumption, as even when the same area is considered, the structure of the problem might be different, as well as the preferences, which might call for a very different method, though the area of work is the same.

### 3.7. Technical support (c.10, phase 3)

The last characteristic that concludes the taxonomy, as well as Fig. 5, is the availability of the software to implement the method(s) as well as its graphical representation and exploration of the results. These features fall outside the objective characterization of MCDA methods capacities, but it still drives the recommendation of MCDA methods. This finding is related to the fact that software tools that implement the MCDA methods and show the results with a variety of customization can help analysts computing the results and showing them to the DM/stakeholders in a structured, compelling and traceable manner [178,32,39]. More specifically, they can enhance the understandability of the decision recommendation by means of advanced exploration of the results and graphical visualization capabilities. These features include, e.g., an explanation which criteria most affect the final results, and which shares of uncertain performance and/or preferences lead to a certain ranking, classification, or choice of the alternatives [85,14]. Additionally, software capable of analyzing turning points in the input data (e.g., thresholds, performances) that can cause a change in the results is another useful feature to help analyst and DM exploring the model and its recommendation [46].

Sensitivity analysis has a natural link with the software that implements the MCDA method. According to which type of analysis the DM desires to conduct, its complexity can vary as it can involve the performances of the alternatives or the DM’s preferences, or both. There are MCDA methods that are implemented in software tools, and the sensitivity analysis is an associated feature, like DECERNS, PROMETHEE-GAIA, Logical Decisions, M-MACBETH, and VISA [103,85,178].

## 4. Clustering of DSSs for MCDA method(s) recommendation

**Fig. 6.**
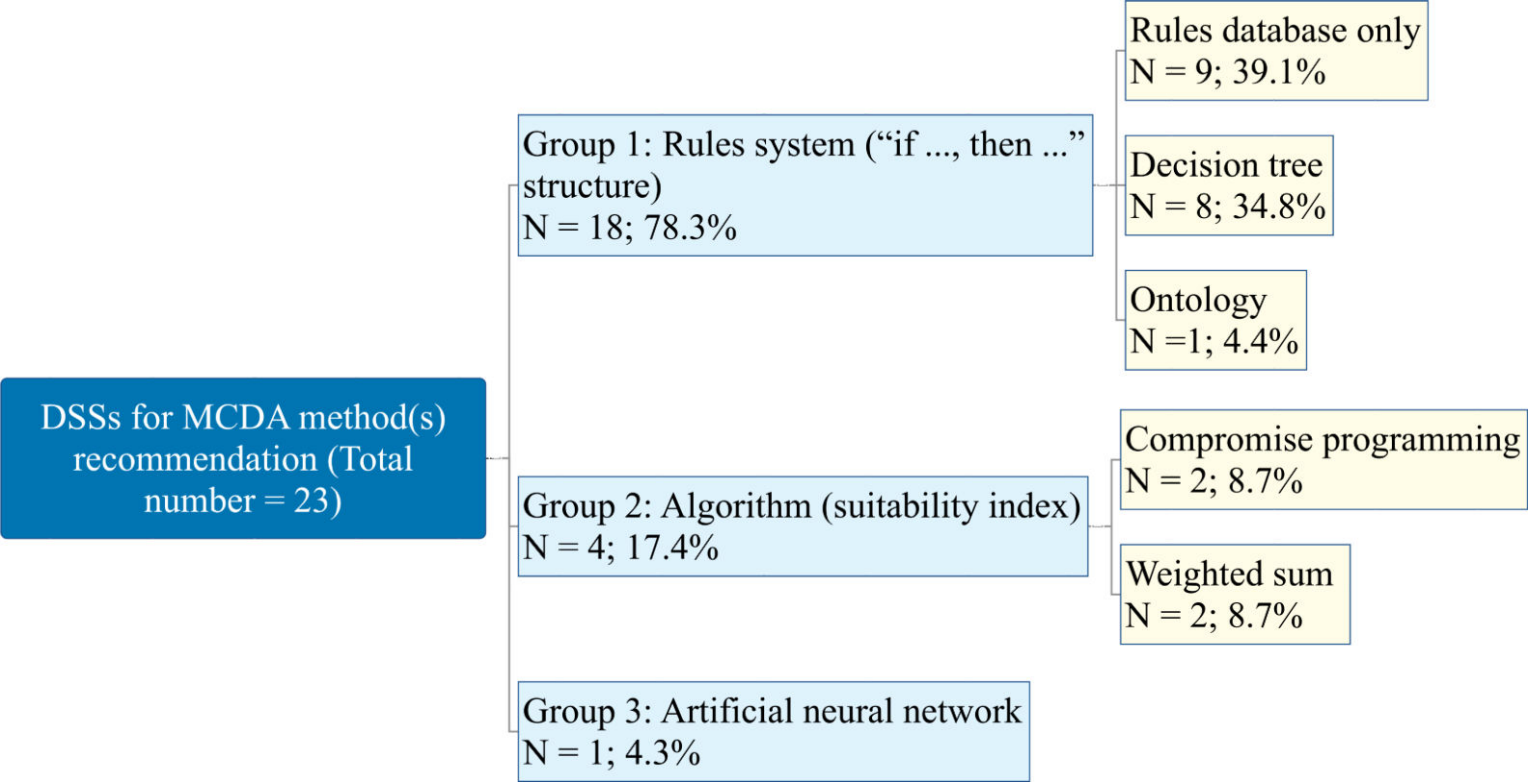
Approaches used for development of DSSs for MCDA method(s) recommendation. *N* = number of studies in each group and% with respect to the 23 in total.

The previous section described the structure of the taxonomy of the MCDA process characteristics and the coverage they received in the reviewed studies. According to their combination, these characteristics can then be used to lead the selection of MCDA methods for each specific decision making problem. This is normally accomplished by DSSs, which in this review accounts for just over 40% of the studies, confirming the wide interest in providing platforms that the analyst can use to lead decision aiding comprehensively. In this section, a grouping of the 23 DSSs that have been used to recommend MCDA methods is proposed. Three main groups have been identified, namely the rules-based, algorithm-based, and artificial neural network-based systems (see Fig. 6).

The first and by far the most commonly used (78.3%) are the rules-based systems, which are grounded on a series of questions that eliminate those methods that do not fit the requirements of the user until the most appropriate one or ones are identified. They are usually based on “if…, then…” knowledge rules [177]. More specifically, rules syntax consists of “*if characteristics a, b, … are activated/met, then MCDA method I (II, …) fit with the problem and can be recommended* ”. An example is “*if weights are importance coefficients, then a non-compensatory method should be used* ”. Half (9) of the rules-based systems directly use the rules in the database to lead the method search, while two groups of more refined systems can be distinguished within this cluster, the decision tree, and the ontology.

**Fig. 7.**
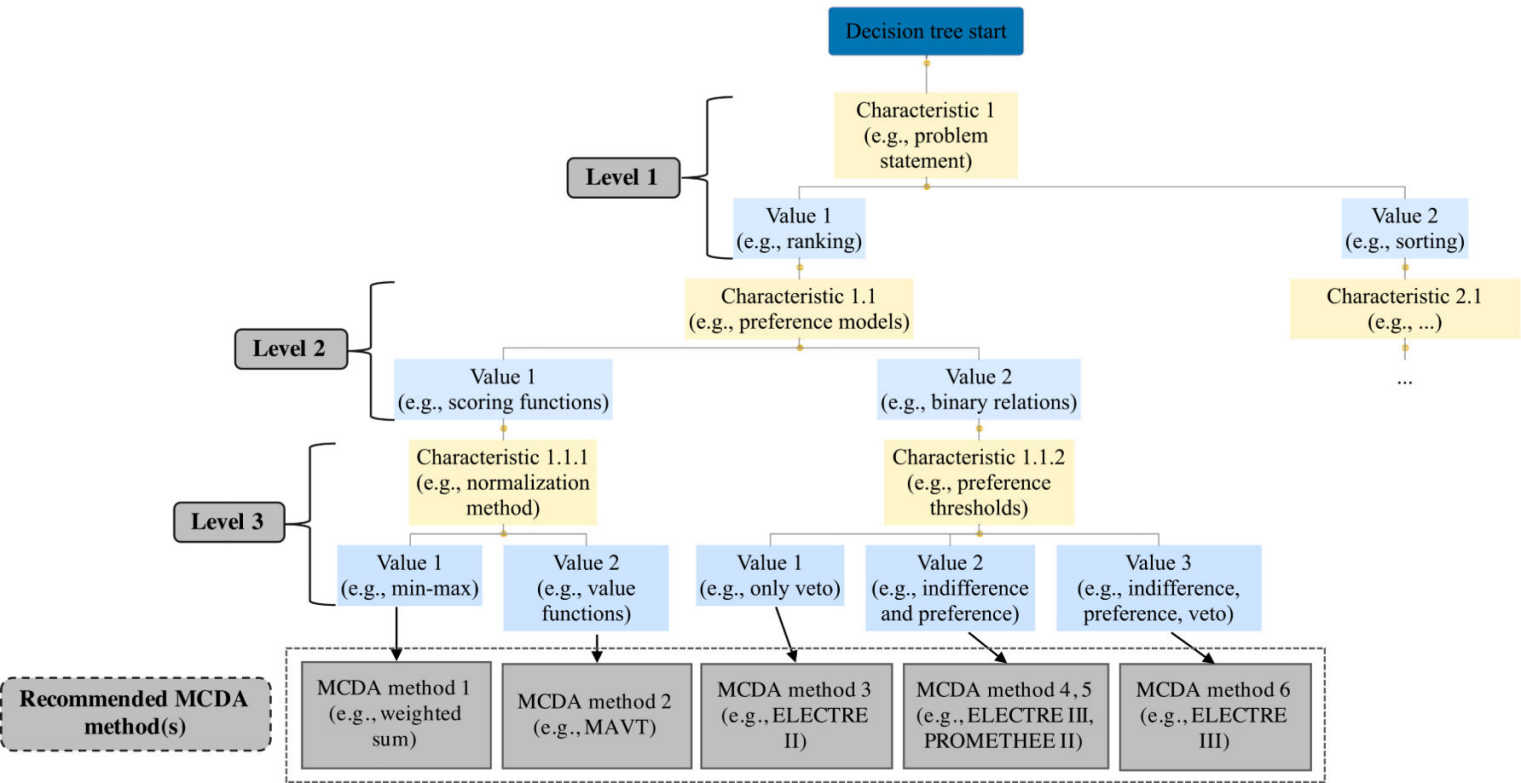
Example of DSS for MCDA method(s) recommendation using a rules system-based decision tree.

As part of the rules-based group, the decision tree has been a traditional approach to MCDA methods selection, based on a taxonomy of the methods that consists of nodes and branches linked by choice rules. The users are led through the tree by narrowing down their options by following the branches, usually responding to a specific question (e.g., do you want to obtain a ranking or sorting of the alternatives?) at each node with respect to each decision support stage. Fig. 7 shows a schematic example of the DSS implementing a decision tree, structured on three main levels. The first one looks at the type of problem statement that the user is interested in, distinguishing between ranking and sorting. The second level is focused on the types of preference models that the user would like the MCDA method(s) to implement, being in this case either scoring functions or binary relations. The last level considers the type of normalization method for the scoring functions branch, and the types of preference thresholds for the binary relations branch. According to the choices of the user, he/she can be recommended a single method (e.g., MAVT) or more than one (e.g., ELECTRE III, PROMETHEE II).

One limitation of the rules-based approaches is that they rarely lead to the identification of a single method, but rather to a set of methods, leaving the selection of the candidate method to the user [130,114,173]. In the case of the tree representation, even when the tree leads to the identification of a single method like in the case of Teghem et al. [160], it is not clear why the questioning for the user is flat and not hierarchical, so that as soon as one condition of a rule is met (e.g., partial-order ranking), a specific method is recommended (i.e., ELECTRE III), even if there would be another in the database that would fit as well (e.g., PROMETHEE I). What is more, due to a large number of MCDA methods, the multiple characteristics needed to differentiate the methods, as well as the extension of more than two branches for some nodes (e.g., problem type), would result in a combinatorial explosion of the nodes. This makes the construction of a tree unpractical and not easy to apply, which partially compromises the purpose of decision aiding [130].

Tiered rules-based approaches have also been proposed, where screening rules are used to eliminate unsuitable methods, while evaluation rules are used to filter the methods to identify the one(s) that best match(es) the described problem (for an example see Ozernoy [129]).

The other group of systems for MCDA method(s) recommendation building upon the rules includes those that use ontologies to map the capacities of MCDA methods to deal with certain decision making problems [174,176]. Ontologies are explicit representations of the concepts and relationships that characterize a certain domain, such as, in this case, the MCDA methods. In addition, ontologies can be queried to determine the relevant method that fits with the selected features. In fact, the object properties are used to match the conditions (“if” part of the rule) with the methods that satisfy them (“then” part of the rule). The added value of the ontological representation of MCDA methods consists in explicitly defining their components as well as the relations between them. It can be seen as an enhanced database representation of methods’ capabilities. So far, only one example of ontology-based DSSs for MCDA method(s) recommendation has been proposed [174,176]. It includes a limited set of methods (i.e., 25) and characteristics, being the compensation effect, the preferences model desired by the DM, the family of aggregation algorithm, and the type of input data.

The second cluster, which represents 17.4% of the analysed DSSs, includes algorithm-based approaches which aggregate the characteristics of the decision making problem and provide an overall indication of the suitability of different methods, which are respectively ranked according to it (e.g., Li et al. [102] and Guarini et al. [76]). The algorithm used can vary from study to study, including compromise programming [64,159] and weighted averages [102,76].

A hybrid approach based on screening rules first to eliminate methods that are considered unfit for the problem and using an algorithm later to define a suitability level for the remaining options has been presented by Gershon and Duckstein [64]. The reason for this structure is that the screening rules consider mandatory characteristics of the problem that the authors think should be necessarily satisfied by the methods, like the management of qualitative criteria, or the ability to handle uncertainty. These characteristics are not considered as biding in the other analyzed DSSs, which shows another avenue of future research to discuss which characteristics are relevant for eliminating methods from a starting group and which ones are not.

A unique example of the third cluster of DSSs for MCDA method(s) recommendation employs an artificial neural network (ANN), which is presented as a notable alternative to circumvent the vicious cycle of using MCDA methods to develop such systems [167]. The ANN was developed by accounting for six features of the MCDA problem, namely (i) the problem statement, (ii) the number of the considered alternatives and criteria, (iii) the type of preferences, (iv) the preference model desired by the DM, (v) the type of measurement scales, and (vi) the weighting of the criteria. The training of the network was conducted with the backpropagation algorithm based on 21 MCDA methods (among which ELECTRE, PROMETHEE, TOPSIS, AHP) that exactly matched the characteristics and tested on a series of decision making problems to check its success rate. The units (neurons) in the input layer were provided with the values on six mentioned features, while the units in the output layer were related to either the clusters of MCDA methods in the first-level testing (including elementary, interactive, value- and outranking-based approaches) or the MCDA methods themselves in the second-level testing. As far as first-level testing was concerned, over 80% classification accuracy was achieved, while for the second-level testing, it was between 63.6% and 73.3%. An overlooked aspect of this work has been a description of how to justify the recommended method if it does not perfectly match the characteristics of the problem. In other words, if, e.g., the user desires a probabilistic ranking, but the only available method in the learned system deals with deterministic rankings, then the recommendation of the latter method might leave the user unsatisfied and possibly reluctant to use the system again.

The review found that the majority of the approaches (78.3%) to recommend the MCDA methods are built on rules, appearing consistently since the 1980s [160,134,175,177]. Their success might be related to the fact that they exploit the factual knowledge on the MCDA methods, building upon the conditions that trigger the suitability of a certain MCDA method. In two rules-based systems proposed by Wątróbski and Jankowski [175] and Wątróbskį et al. [177] it was notable to see that the rules validation was conducted by looking at whether the MCDA methods recommended were the same as those used in the existing case studies, a finding that was eventually confirmed in the majority of cases.

Only one study reported a survey of DSSs for MCDA method(s) selection [96]. This study looked at whether six approaches to lead the selection of MCDA methods considered or not several characteristics, among which were the type of decision problematic, number of alternatives, presence of weighting, and measurement scale. Even though the research shows a notable cover of key features of decision aiding, the authors emphasize the need for MCDA selection approaches that are application-independent, cover more MCDA methods and also a broader set of problem types.

The algorithm-based approaches that recommend an MCDA method require (or at least allow) the setting of weights for each characteristic to run the selection algorithm [64,159,102,76]. This adds a layer of subjectivity that clashes with the goal of being as objective as possible in the selection of the MCDA method. Furthermore, the selection of the algorithm itself entails subjectivity, which causes a closed loop (defined also as vicious circle by Guitouni and Martel [78]) where the solution to the problem is found through an arbitrarily chosen/developed algorithm, raising the question on impartiality of the proposed approach, a consideration confirmed by one of the authors of such DSS [159].

The approach that can be seen as less subjective and as more impartial is the rules-based one. This representation includes “if…, then…” rules that are used to explain MCDA methods as well as to recommend them. Such an approach has been used for several decades [130,134] and is still used in state-of-the-art DSSs, like the one presented by Wątróbskį et al. [177]. However, the subjectivity is also driven by the nature of the characteristics the systems work with, though here the focus is on the differences between the implications of the working process of the recommending systems.

## 5. How to apply the proposed taxonomy to describe MCDA methods and identify the appropriate one(s) for a specific case study?

The taxonomy proposed in this paper provides a tool to assess MCDA methods according to what type of problem they can be suited for. It empowers the analysts to select the MCDA method(s) that best fit the decision making problem at hand. The intended users of this taxonomy are thus decision analysts, whose responsibility is to conduct an evaluation of a set of alternatives, assessed on multiple criteria, using MCDA methods, and to support the DMs in the MCDA process.

In this section, we discuss an approach to make the taxonomy operational. It builds upon the rule-based type of DSS (Group 1) presented in Section 4. The selection of this reasoning system is driven by the simplicity and understandability of the decision rules. The taxonomy, with its characteristics, can be used to conduct a mapping of the MCDA methods, as well as the case studies. In this case, “mapping” is interpreted as the evaluation of which (sub-) characteristics of the taxonomy (i) are supported by the MCDA methods and (ii) are relevant to describe each case study. Example questions that can be used to map the MCDA methods and describe a case study are presented in Table 2. They refer to specific (sub-)characteristics of the taxonomy, in this case, c.1.1 = problem statement, c.1.2 = set of alternatives, and c.2.1 = structure of the criteria.

To further demonstrate, the application of each of these questions to two MCDA methods and two case studies is shown in Table 3. The upper portion of the table highlights the first method supporting sorting problems as well as the second method tailored to ranking. As far as the set of alternatives is concerned, the first method can accept both stable and unstable problem contexts, while the second method can only address those that are stable. Lastly, both methods 1 and 2 can accommodate flat structures of criteria, whereas problems with hierarchical criteria can only be managed by method 2. The lower portion of the table presents a description of two case studies, showing which type of problem statement they impose, whether the set of alternatives is stable or not, and the structure of the criteria. At this point, the identification of the appropriate MCDA method(s) for each case study is determined by the best match of the mappings in each row (see the last column in Table 3). The first case study leads to method 1, and the second case study results in method 2. In other words, the challenge consists in evaluating which (sub-) characteristics of each case study are also supported by the relevant MCDA method(s).

Tables 2 and 3 present a simplified example of the application of the taxonomy. To fully implement and conduct a mapping of the MCDA methods, a complete questioning strategy is needed and proposed below:

1. What is the supported problem statement?
2. Does the method support a set of stable and/or incremental alternatives?
3. Can the method accept a flat and/or hierarchical structure of the criteria?
4. What is the measurement scale that can be used (in a mathematically meaningful manner)?
5. Can deterministic and/or uncertain criteria performances be accepted?
  - If uncertain performances are acceptable, of what type(s) can they be?
6. If weights can be used, are they subjective and/or objective?
  - If they can be subjective, are they precise and/or imprecise?
    - If they are precise, are they trade-offs or importance coefficients?
    - If they are imprecise, of what type can they be?
  - If they can be objective, of what type(s) can they be?
7. If thresholds can be used, of what type can they be?
8. Can interactions between criteria be included?
9. What type of preference model is used to aggregate the input data?
10. If indirect preference information can be used, what is the frequency of preference provision and the supported elicitation approach(es)?
11. What is the level of compensation used in the aggregation algorithm?
12. Can inconsistent preferences be included?
13. Is the method prone to rank reversal?
14. Should the preference model be exploited by developing a univocal recommendation or by looking at its robustness?
15. Can group decision making be supported?
16. How easy is it to understand and use the method?
17. What is the processing time needed to compile the data required for the method?
18. What is the number of alternatives and/or criteria the method can work with?
19. What is the extent of the use of the method in a specific context/area?
20. Is there software available to use the method?
  - If the software is available, how is its graphical representation capability?

The answers to these questions are those available values in Table 1 in the most-right position before the frequency column. To show the applicability of this approach, a subset of 16 MCDA methods were selected from literature and mapped. Some well-known methods were selected, including the AHP, ELECTRE I and III, MAVT, PROMETHEE II, DRSA for sorting, TOPSIS, and revised VIKOR. Extensions of methods were also included to show the ductility of the taxonomy, with the selection of SMAA-PROMETHEE, and several variants of TOPSIS. As far as the latter is concerned, the variants account for different types of performance of alternatives and preferences of the DM (i.e., classical, interval, fuzzy) and their combination (i.e., interval and fuzzy). In addition, the distinction between single and group decision making is included. Appendix C in the ESI shows the application of the taxonomy on this set of MCDA methods.

According to the current structure of the taxonomy, the questions for Phases 1 and 2 (i.e., questions from 1 to 15) allow the analyst to provide a rather unambiguous answer to each question. The characteristics in these phases deal with features the MCDA methods can and/or should be clear whether they can support or not. On the other hand, Phase 3 involves more qualitative characteristics, and their answers can be subject to the interpretation of researchers. As a result, the same method might be classified differently by various researchers [152]. In this case, the proposed solution is to either ask the method’s developers for the desired value or adopt the predominant view in the literature.

To apply the proposed questioning strategy in real case studies, the analyst could test it on a subset, by adapting the wording of each question to describe each decision problem, as shown by the examples in Tables 2 and 3. Such applications constitute a promising avenue for future research.

## 6. Discussion and conclusion

Supporting decision making is a complex task as it requires a multitude of competencies and skills that include problem formulation, mathematical modeling, programming, dialectic, and project and risk management, among others. Over the last few decades, researchers and practitioners from operational research, decision, and information science areas have proposed frameworks, approaches, and DSSs that can be used to aid in decision making. Building upon, and to maximize their efforts, this paper has proposed a taxonomy of the MCDA process characteristics. This work represents the first stage of a contribution towards the harmonization phase for MCDA consisting of a structured and comprehensive representation and definition of the components that should be considered when leading multiple criteria-based decision aiding. This taxonomy was derived from a review of the available literature that provided guidelines and approaches to conduct the MCDA process, to choose an MCDA method or a subset of methods, or that discussed the strengths and weaknesses of MCDA methods.

By segmenting the MCDA process in three main phases, (i) problem formulation, (ii) construction of the decision recommendation, and (iii) qualitative features and technical support, the taxonomy shows how decision making can be split into manageable steps and components, also called characteristics. This allows analysts, as well as the DMs/stakeholders, to avoid the risk of being overwhelmed by the entity of the task.

The main distinguishing factor of the proposed taxonomy when compared to previous ones is the coverage of characteristics. The one proposed in this paper is the most comprehensive so far, including the different components of the MCDA process. The authors found that the previous taxonomies presented in the literature focused on a limited set of characteristics. In addition, the available taxonomies looked at the conduct of the MCDA process from a rather high-level perspective, without going much in detail with respect to each characteristic and its possible diversification (i.e., values). An example is the use of uncertain performance of the alternatives, where the majority of the studies looking at this feature mentioned the use of probabilistic and fuzzy approaches as the main examples. However, there are several other strategies (e.g., evidential reasoning, possibility theory, gray numbers) to deal with the uncertainty of input data, which just two publications extensively considered (i.e., Dias et al. [43] and Pelissari et al. [132]). Another example is the type of weights that can be used. A majority of the publications looking at weighting identify the precise subjective weights, being either tradeoffs (45%) or importance coefficients (36%). Those looking (also) at the other type of subjective weights (i.e., imprecise) just mention their existence, and only a limited number of publications provide their specific typologies (e.g., Riabacke et al. [140]). We found that the additional level of detail in the specific publications was justified by the fact that the publications were specifically focused on that topic and provided less attention to other parts of the MCDA process. These differences highlight the distinctive character of the taxonomy presented in this paper. Even if it provides comprehensive coverage of the characteristics that constitute the MCDA process, it also offers a detailed overview of each of them, together with its sub-characteristics and possible values.

### 6.1. Research gaps

#### 6.1.1. Phase 1: problem formulation

The selection of the problem statement is the first main decision that has to be undertaken during the problem formulation stage. Ranking, choice, and sorting are the main types mentioned in the reviewed publications. A research gap that emerged is that more attention should be devoted to differentiating MCDA methods according to their capacity to deal with different types of orders of the alternatives (i.e., partial, complete) and the type of the recommendation (i.e., ordinal or cardinal). These important subdivisions have only been discussed in some publications, like Roy and Słowiński [148], Maggino [104], Celik and Topcu [27], Greco et al. [73], Tsoukiàs et al. [166], and Munda [126]. They deserve more attention in future research as they have such a notable effect on the type of MCDA method that suits the decision making challenge. Another aspect that is missing in the reviewed publications concerns cardinality constraints on the provided recommendation. They may be associated with choice [133] or sorting [91] problems to delimit the number of alternatives that are selected or assigned to a particular class, respectively.

The increasing complexity of MCDA problems, especially related to the growing number of criteria, has shown that their hierarchical structuring improves problem formulation and communication between stakeholders and DM [111]. This review found that the DSSs that recommend MCDA method(s) lack attention to this important capability, which indicates a valuable avenue for future research. In addition to the structure of the criteria, its number should also have an effect on the relevant MCDA method. Nonetheless, this is a rarely considered topic. On one hand, for ELECTRE methods, there is a general recommendation that they should be used when the number of criteria is at least three and not more than twelve or thirteen [58]. On the other hand, the cognitive burden of indirect questions used in preference disaggregation approaches is also confirmed to depend on the number of criteria. As a result, holistic questions (e.g., concerning pairwise comparisons or assignment examples) with up to six criteria are usually deemed feasible for DMs to answer [150,34]. The review shows that for other methods, there are no such recommendations, which indicates a knowledge gap that should be filled by future literature proposing new MCDA methods.

A partially overlooked topic is the fit between the type of measurement scale of the criteria and the selected aggregation function (part of preference model), precisely the way this data is used by the function to derive the decision recommendation. Many arbitrary choices (e.g., choice of numerical coding, equivalence of intervals between the ordinal scale) are made when converting ordinal scales to cardinal ones, a primary requirement for score-oriented methods [19,126,118,104,117]. In these cases, it would be preferable to use methods that can work directly with such type of scale, without any need for transformations, for example with outranking or decision rules [135,148]. What is more, there are methods that are particularly suited to problems when there is a strong heterogeneity related to the nature of evaluation criteria, which makes it difficult to aggregate all criteria in a unique and common scale. In these cases, methods that either exploit only the ordinal properties of the scales (e.g., DRSA) and compare alternatives pairwise on the individual criteria using the original scales (e.g., ELECTRE or PROMETHEE) are particularly suited. The DSSs would aid a more mathematically consistent use of the raw data if these considerations were included in their future versions.

#### 6.1.2. Phase 2: construction of the decision recommendation

Weighting is an essential step in the MCDA process, and it has been studied in several of the reviewed publications. An issue that this review highlighted is that there is still a lack of alignment between the type of precise weights (either trade-offs or importance coefficients) and the aggregation algorithm that should be used with each of them. More specifically, trade-offs should be used with scoring functions, while importance coefficients with outranking algorithms [126]. This methodological requirement is missing in the surveyed DSSs. This is an important indication of possible mathematical inconsistency when weights of a specific type, like importance coefficients, are used with MCDA methods that require the other type of precise weights, e.g., when using weighted sums [127,116]. Also, the use of weights as trade-offs (“gain with respect to one variable allowing to compensate the loss with respect to another” [127]) implies a direct link with the measurement scale. Consequently, if one is changing, the other has to change accordingly [142]. We found that, due to their simplicity, simpler weights elicitation techniques are sometimes used instead of more demanding ones [118]. These considerations should receive further attention to guarantee consistent use of weights in MCDA methods.

Interactions between criteria should be a key avenue of further focus, because of the recurrent recognition of the need to account for the interrelations and dependencies between criteria [11,60]. Although only six (among which only one DSS) of the 56 studies reviewed in this paper consider the interactions between criteria as a distinctive feature, several MCDA methods dealing with interactions have been proposed [68,69,8], particularly in recent years [7,20,36,5]. The growing interest in handling interactions indicates that DSSs recommending MCDA methods would benefit from including this discriminatory characteristic.

There are also several modeling assumptions that are not tested in practice when selecting MCDA methods. For example, Montibeller et al. [117] found that in multiple studies dealing with health threats prioritization, several requirements for the application of value-based MCDA methods were not met or at least explored by the authors. These included the preferential independence among criteria, the shape of the value functions, and the weights as trade-offs. It thus appears very important that future studies should conduct axiomatic analysis and elicit preferences with adequate protocols to avoid producing biased results that can lead to mathematically-unfounded decisions.

As far as preference elicitation is concerned, indirect methods are receiving increasing attention because they do not require such extensive and demanding interaction with the DMs as direct methods do. They operate based on natural human reasoning that can also be provided interactively, in the form of local or comprehensive statements [157]. Overall, given the ductility, as well as the limited effort required from the DM when using MCDA methods, it seems sensible to recommend that further focus and energy should be placed on the development of such approaches. This research avenue could contribute to providing objective solutions for dealing with the critiques on MCDA as being laborious in terms of preferences elicitation [71], which might sometimes prevent their use in real-life decision contexts. It must, however, be noted that one drawback of indirect MCDA methods is their mathematical complexity, which makes them sometimes more difficult to apply by the analysts than the direct ones [52].

Management of inconsistent preferences has received limited attention in the reviewed studies. It is though well known that the same DM might be inconsistent in his/her judgments, or the group providing the preferences may not unanimously agree with the same preferences [48,155]. As a consequence, it is desirable to have more MCDA methods that can deal with these types of decision context due to their inbuilt features. Another solution would be to have more general frameworks for dealing with inconsistency in preference information that can be coupled with different MCDA approaches [121,119].

The concept of compensation appears to be embedded in the majority of the literature. It indicates a strong understanding of the possibility of matching the value system of the decision makers with the aggregation algorithm that has the desired compensatory level. It is thus essential for future MCDA methods and DSSs recommending to include this critical discriminatory feature, to select an appropriate method with a desirable trade-off level between criteria [56].

The rank reversal problem is still largely unexplored for many MCDA methods, as confirmed by the recent review by Aires and Ferreira [2]. The future focus should be directed towards the explanation of its causes and also to the proposal of convincing solutions to communicate it to the DM. A closely related topic that has not received attention in the MCDA literature consists in studying the possible modifications of the best choice alternatives or class assignments for problems involving cardinality constraints [133,91].

Finding solutions to add legitimacy to decision recommendations from MCDA methods is still a notable challenge for the research community. Most of the applications of MCDA methods have not been validated and their results are mostly based on a single model that provides deterministic results based on deterministic performances and preferences of the DM. As confirmed by Katsikopoulos et al. [94], this “ground-truth” does not objectively exist in MCDA, because the evaluation of the alternatives requires the inclusion of the preferences of the DM, which means that the final recommendation can change according to these preferences. This is the main difference of preference (i.e., MCDA) models when compared to inference and forecasting models [38]. In the latter case, the exact answer is known, and the challenge is to mathematically combine the attributes to predict or forecast the outcome reasonably well. As far as preference models are concerned, Hogarth and Karelaia [82] found that decision support strategies using simple models provide the same results as those “expected” when assuming a more complex one (e.g., linear multi-attribute). The authors found that using binary criteria, models looking at the ordinal positions of the alternatives would provide in the significant (if not all) share of the cases, the same results as those obtained with a more complex linear multi-attribute model. This topic has also been looked at by Katsikopoulos [93], who found that in several cases, simpler preference models performed as good (if not better) than more complex ones. If in MCDA we consider a complex model that requires many inputs from the DM and cognitive effort, it would be possible to consider the use of a method that can be more easily understood and with less demanding input [94]. Some examples would be to use imprecise weights rather than exact trade-offs, such as intervals or ordinal rankings. These findings raise the question “*How complex should MCDA methods be to provide decision support, while at the same time being understandable by the DM?*”.

However, increasing the legitimacy of the results is also possible by looking at robustness and sensitivity analysis. Robustness analysis allows studying the variability of the results according to all or most plausible sets of performance or the preference model parameters compatible with the preference information. It is interesting to notice that a broad spectrum of recent MCDA methods has currently been developed with these capabilities. Some examples include Corrente et al. [38] and [37], Kadziński et al. [89], Greco et al. [72], Angilella et al. [6], and Kadziński and Ciomek [88], confirming the growing attention of the MCDA community to the issue of legitimization of the decision recommendation. Another solution to test results legitimacy is to exploit sensitivity analysis, which can be used to study if and how the recommendation already derived with a particular method would be affected under different alternatives performance and/or DM’s preferences. Examples in this regard are the works by Ferretti et al. [54] and Ciomek et al. [33], where sensitivity on weighting and performances, respectively, is used to evaluate the stability of the best alternatives, attained ranks, or pairwise preference relations.

An interesting avenue for future research consists in proposing a set of detailed features for specifically describing group decision methods. We have identified the following characteristics serving this purpose: the level on which individual preferences are aggregated, admitting different roles and/or importance degrees of the involved DMs, and the number of stakeholders whose preferences can be handled. In particular, the suitability of MCDA methods to be used in the participatory processes vastly depends on the skills of the analyst and the software support. However, some peculiar features of different approaches imply that they are applicable in scenarios involving a high number of stakeholders, potentially representing various interest groups. These characteristics include the aggregation of preferences at the output level, the capacity of dealing with inconsistencies, the short processing time needed to compile data [100], and “easiness” of the underlying preference elicitation protocol without oversimplifying the problem [161].

#### 6.1.3. Phase 3: qualitative features and technical support

The majority of the characteristics in the taxonomy are objective features. They do not depend on the decision support training of the analyst and/or the mathematical literacy as well as the requirements of the DMs/stakeholders. There are, however, studies that look at qualitative elements while searching for MCDA methods, including the easiness of use, the extent of use of the methods in the specific context, and the availability of the method in software. These characteristics do not usually have an associated objective level of attainment, which obliged the authors to develop evaluation scales (mostly ordinal) on their own [129,159,135,25,112,148,31,76]. This is the main point to be considered when developing DSSs aimed at recommending MCDA methods in an unbiased mode, or at least as objectively as possible.

The availability of software implementing MCDA methods has also had a notable effect on the use of certain methods. It is, in fact, not uncommon that analysts look for software that implements the selected approach [178]. This is especially the case in projects that lack personnel with the requisite programming skills. There might be, however, cases where there is no available software that implements the desired MCDA method and/or the sensitivity analysis as well. In such cases, the analyst has to find a solution either by self-coding or outsourcing its development in a software shell. This is especially the case for newer MCDA methods, which are more advanced than the previous ones and can deal with more complex problem structures and DM’s preferences. Software development is a demanding (cost- and time- wise) activity. As a result, many methods are not implemented in self-standing tools, which can humper their applicability in several areas.

### 6.2. Practical implications for DSS development

The taxonomy of the MCDA process characteristics proposed in this paper can be used to support the development of future DSSs for MCDA method(s) recommendation. Its added value resides in the comprehensiveness as well as modularity of the characteristics. Furthermore, the clustering of DSSs for MCDA method(s) recommendation that was proposed in this paper can be a starting point for a traceable and categorizable development of systems that can help analysts during their decision support activities. Out of the rules-, algorithm- and neural network-based DSSs groups, the former has been the most used one. More specifically, as part of the rules-based group, the ontology-driven systems are a promising avenue of future research for these DSSs. They would allow developing a harmonized and machine-readable vocabulary for the MCDA process and methods characteristics, allowing researchers to more efficiently develop, discuss, and communicate information about their methods as well as decision making per se.

Given the confirmed links and synergies between MCDA and artificial intelligence [47], it would be interesting to see how new models based on ANN would perform with a wider set of decision characteristics and MCDA methods. ANNs have the potential to automatically differentiate the impact of individual features as well as their combinations when deciding on the most relevant MCDA approach. The authors of the sole ANN-based DSS for method(s) recommendation reported difficulties in the learning phase due to the inefficiency of the backpropagation algorithm applied to deep ANNs [167]. However, this system was developed two decades ago, and more efficient algorithms for training ANNs have already been proposed. Their incorporation within a more comprehensive DSS could also contribute to addressing the challenge of validation of the chosen method and would add to testing the cross-discipline fertilization.

One of the main difficulties of this research has been the mapping of the characteristics presented in the systems to recommend the MCDA methods, into the taxonomy when very limited information on such features was presented in the respective studies. Sometimes, the description of the characteristics is mostly limited to a graphical representation (e.g., Laaribi et al. [97,134] and Eldrandaly et al. [52]) or a synthetic definition in a table (e.g., Ozernoy [129], Salinesi and Kornyshova [153] and Li et al. [102]), thus making the filling of our taxonomy more challenging. This finding calls for a more thorough and transparent selection and description of the characteristic to be used in future systems for MCDA method(s) recommendation.

### 6.3. Limitations and suggestions for future research

The (sub-) characteristics of the taxonomy are the building blocks of several disciplines when decisions among several alternatives have to be made based on multiple criteria. This is the case in, e.g., engineering design and management, materials development, energy and transportation systems, policy making, and land remediation [103,14,86,138]. A current limitation of the proposed taxonomy is the lack of testing in actual case studies, as it has only been presented conceptually. A promising avenue of research would thus be to conduct a systematic mapping of this taxonomy with respect to a group of case studies reported in these (and other) application areas. This would allow evaluating how well the characteristics can be used to describe each case study. In addition, it would be possible to understand which MCDA method(s) would be suitable for each case study and/or whether the MCDA method(s) used did match with the description of the problem.

The review is also limited in exploring the links of the taxonomy and its characteristics to the cognitive psychology and behavioral operational research domains. This could be an interesting area to explore, with several studies already available in the context of compensatory and non-compensatory methods. For example, Katsikopoulos et al. [95] reported the link between the type of cues used in heuristics and the effect of compensation, stating that high accuracy can be achieved if the structure of cues is non-compensatory. This strategy is the basis of lexicographic heuristics as well as non-compensatory MCDA methods. This topic was also studied by Hogarth and Karelaia [82], who explored a broad set of compensatory and non-compensatory modeling settings. The authors evaluated the percentage of correct and incorrect (where correct is defined as being consistent with the specification of preferences) choices by different models. They found that simple ones, using the deterministic elimination of alternatives based on the relative weights of the criteria, reach high levels of performance (up to 100% in some cases), especially in cases of non-compensatory preferences. The latter findings were also confirmed by Katsikopoulos [93]. The author further showed that heuristics methods like lexicographic algorithms could outperform complex mathematical models in some prediction exercises. A decision hierarchy was also proposed to choose between heuristics and more complex algorithms, according to the availability of information and the linearity of the DM’s preferences. These findings raise questions as to whether simpler methods can be used to help DM expressing preferences (e.g., trade-offs, preference thresholds) with greater precision and possibly lower cognitive burden.

Lastly, as also recommended by Katsikopoulos et al. [94], more emphasis should be placed on the topic of evaluating the MCDA methods in practice, which is not addressed in the current review. Even though it is objectively impossible to assess whether the decision taken is right or wrong, it would at least be desirable to evaluate the *practical impact of the MCDA methods and their short-comings.* This impact could be referred to as the concept of “empirical evaluation” of DSS, as described in Rhee and Rao [139], focusing on the improvement of the decision quality and speed with the use of the MCDA method. This is usually achieved by controlled experiments with and without the users of such tools.

Overall, this taxonomy can provide a solid foundation for the development of comprehensive DSSs that help an analyst in choosing an MCDA method, responds positively to the needs of the DM, and will satisfy all the constraints characterizing the decision situation.

## Supplementary Material

Supplementary Material Cover Page

Appendix A

Appendix B

Appendix C

## Figures and Tables

**Table 1:** Taxonomy of the MCDA process characteristics (c.) and sub-characteristics (c.x.), frequency and% with respect to the 56 reviewed studies. See bottom of the tables for acronyms.

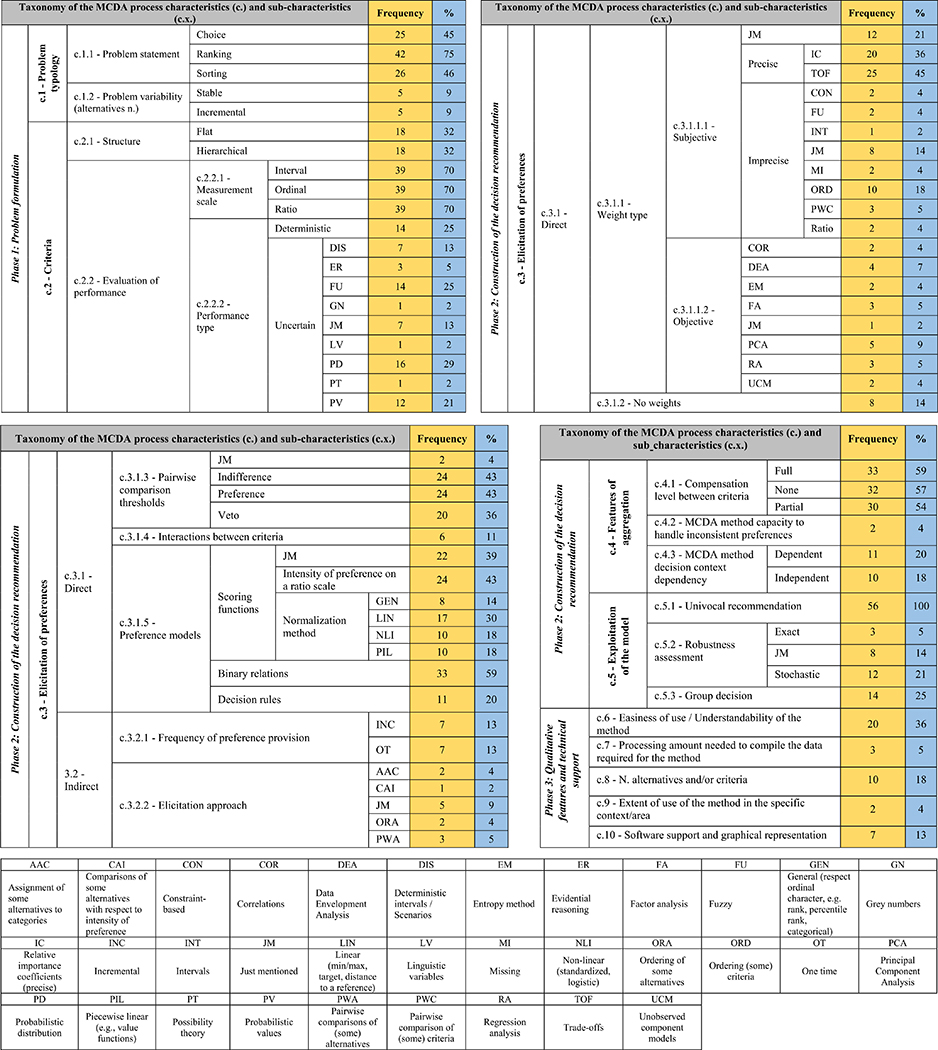

**Table 2:** Example questions used to map the MCDA methods and describe a case study.

Question	Questions to map the MCDA method	Questions to describe the case study
1 for c.1.1	What is the supported problem statement?	What is the problem statement tackled in this project?
2 for c.1.2	Does the method support a set of stable and/or incremental alternatives?	Is the set of alternatives stable and/or incremental?
3 for c.2.1	Can the method accept a flat and/or hierarchical structure of the criteria?	Is the structure of the criteria flat and/or hierarchical?

**Table 3:** Example of mapping of MCDA methods and case studies according to a sample of the (sub-) characteristics of the taxonomy. ✓ in “Mapping of the MCDA methods” = the method supports this (sub-) characteristic; ✓in “Mapping of the case studies” = the case study requests this (sub-) characteristic. The last column shows the selection of the relevant method for each case study.

	c.1 - Problem typology					c.2 - Criteria		Other characteristics	MCDA method(s) suitable for the case study
	c.1.1 - Problem statement			c.1.2 - The set of alternatives		c.2.1 - Structure		...	
	Ranking	Sorting	Choice	Stable	Incre-mental	Flat	Hierar-chical		
**Mapping of the MCDA methods**									
Method 1		✓		✓	✓	✓			
Method 2	✓			✓		✓	✓		
**Mapping of the case studies**									
Case study 1		✓			✓	✓			Method 1
Case study 2	✓			✓			✓		Method 2

## Footnotes

1 We include these approaches in our review because they may be found in several studies. However, they assume that the differences between successive grades are the same, which implies more than what the ordinal scales actually allow to do.
