# Supplementary material for: How to support the application of multiple criteria decision analysis? Let us start with a comprehensive taxonomy
Source: Omega. Author manuscript; Available in PMC 2021 Mar 18. (PMC7970504; doi:10.1016/j.omega.2020.102261)
Supplement: Supplementary Material Cover Page [file NIHMS1673395-supplement-Supplementary_Material_Cover_Page.docx]

**Electronic Supplementary Information (ESI)**

*for the paper*

How to Support the Application of Multiple Criteria Decision Analysis?

Let Us Start with a Comprehensive Taxonomy

Marco Cinelli^1,^,*^, Miłosz Kadziński^1^, Michael Gonzalez^2^, Roman Słowiński^1,3^

*^1^ Institute of Computing Science, Poznań University of Technology, Piotrowo 2, 60-965 Poznań, Poland (*[*marco.cinelli@put.poznan.pl*](mailto:marco.cinelli@put.poznan.pl)*;* [*milosz.kadzinski@cs.put.poznan.pl*](mailto:milosz.kadzinski@cs.put.poznan.pl)*;* [*roman.slowinski@cs.put.poznan.pl*](mailto:roman.slowinski@cs.put.poznan.pl)*)*

*^2^ Environmental Decision Analytics Branch, Land Remediation and Technology Division, Center for Environmental Solutions and Emergency Response, Office of Research and Development, U.S. Environmental Protection Agency, 26 West Martin Luther King Dr., Cincinnati, 45268, Ohio, USA (*[*gonzalez.michael@epa.gov*](mailto:gonzalez.michael@epa.gov)*)*

*^3^ Systems Research Institute, Polish Academy of Sciences, Newelska 6, 01-447 Warsaw, Poland*

^ Present address: Environmental Decision Analytics Branch, Land Remediation and Technology Division, Center for Environmental Solutions and Emergency Response, Office of Research and Development, U.S. Environmental Protection Agency, 26 West Martin Luther King Dr., Cincinnati, 45268, Ohio, USA

^*^ Corresponding author

**Appendix A – Publications used to develop the taxonomy (see respective Excel file)**

Mapping of the publications (in alphabetic order) that met the inclusion requirements for the literature review and that were used to develop the taxonomy of the MCDA process (sub-)characteristics. They are divided in two groups: Group 1. Publications supporting (part of) the MCDA process and comparing MCDA methods (N = 33); Group 2. DSSs recommending a specific MCDA method or a subset of MCDA methods (N = 23). The characteristics of the taxonomy are reported from column D onwards, where the symbol ✓ indicates that a publication considered the characteristic.

**Appendix B – Description of the MCDA process (sub-)characteristics (see respective Excel file)**

Brief description of each (sub-)characteristic that constitute part of the taxonomy.

**Appendix C – Mapping of 16 MCDA methods according to the (sub-)characteristics of the taxonomy (see respective Excel file)**

Application of the taxonomy on a set of 16 MCDA methods. Well known methods were selected, including the AHP, ELECTRE I and III, MAVT, PROMETHEE II, DRSA for sorting, TOPSIS and revised VIKOR. Extension of methods were also included to show the ductility of the taxonomy, with the example of SMAA-PROMETHEE, and several variants of TOPSIS. As far as the latter is concerned, the variants allow to account for different types of performance of alternatives and preferences of the DM (i.e., classical, interval, fuzzy) and their combination (i.e., interval and fuzzy). In addition, distinction between single and group decision making is included too.
